# A Direct-to-Public Peer Support Program (Big White Wall) Versus Web-Based Information to Aid the Self-management of Depression and Anxiety: Results and Challenges of an Automated Randomized Controlled Trial

**DOI:** 10.2196/23487

**Published:** 2021-04-23

**Authors:** Richard Morriss, Catherine Kaylor-Hughes, Matthew Rawsthorne, Neil Coulson, Sandra Simpson, Boliang Guo, Marilyn James, James Lathe, Paul Moran, Laila J Tata, Laura Williams

**Affiliations:** 1 Institute of Mental Health University of Nottingham Nottingham United Kingdom; 2 Department of General Practice University of Melbourne Melbourne United Kingdom; 3 School of Medicine University of Nottingham Nottingham United Kingdom; 4 Research Delivery Team Nottinghamshire Healthcare NHS Foundation Trust Nottingham United Kingdom; 5 Centre for Longitudinal Studies University College London London United Kingdom; 6 School of Medicine University of Bristol Bristol United Kingdom

**Keywords:** peer support, digital mental health, depression, anxiety, population reach, productivity, mobile phone

## Abstract

**Background:**

Effective help for depression and anxiety reaches a small proportion of people who might benefit from it. The scale of the problem suggests the need for effective, safe web-based public health services delivered directly to the public. One model, the Big White Wall (BWW), offers peer support at low cost. As these interventions are delivered digitally, we tested whether a randomized controlled trial (RCT) intervention could also be fully delivered and evaluated digitally.

**Objective:**

This study aims to determine the reach, feasibility, acceptability, baseline costs, and outcomes of a public health campaign for an automated RCT of the BWW, providing digital peer support and information, compared with a standard website used by the National Health Service Moodzone (MZ), to people with probable mild-to-moderate depression and anxiety disorder. The primary outcome was the change in self-rated well-being at 6 weeks, measured using the Warwick-Edinburgh Mental Well-Being Scale.

**Methods:**

An 18-month campaign was conducted across Nottinghamshire, the United Kingdom (target population 914,000) to advertise the trial directly to the public through general marketing, web-based and social media sources, health services, other public services, and third-sector groups. The population reach of this campaign was examined by the number of people accessing the study website and self-registering to the study. A pragmatic, parallel-group, single-blind RCT was then conducted using a fully automated trial website in which eligible participants were randomized to receive either 6 months of access to BWW or signposted to MZ. Those eligible for participation were aged >16 years with probable mild-to-moderate depression or anxiety disorders.

**Results:**

Of 6483 visitors to the study website, 1510 (23.29%) were eligible. Overall, 790 of 1510 (52.32%) visitors participated. Of 790 visitors, 397 (50.3%) were randomized to BWW and 393 (49.7%) to MZ. Their mean age was 38 (SD 13.8) years, 81.0% (640/790) were female, 93.4% (738/790) were White, and 47.4% (271/572) had no contact with health services in the previous 3 months. We estimated 3-month productivity losses of £1001.01 (95% CI 868.75-1133.27; US $1380.79; 95% CI 1198.35-1563.23) per person for those employed. Only 16.6% (131/790) participants completed the primary outcome assessment. There were no differences in the primary or secondary outcomes between the 2 groups.

**Conclusions:**

Most participants reached and those eligible for this trial of digital interventions were White women not in recent contact with health services and whose productivity losses represent a significant annual societal burden. A fully automated RCT recruiting directly from the public failed to recruit and retain sufficient participants to test the clinical effectiveness of this digital intervention, primarily because it did not personally engage participants and explain how these unfamiliar interventions might benefit them.

**Trial Registration:**

International Standard Randomized Controlled Trial Number (ISRCTN) 12673428; https://www.isrctn.com/ISRCTN12673428

**International Registered Report Identifier (IRRID):**

RR2-10.2196/resprot.8061

## Introduction

### Background

Depression and anxiety are, respectively, the first and sixth leading causes of years lived with disability globally among all health problems [1]. In the United Kingdom in 2014, 15% of the general population of adults aged >18 years had depression or anxiety, but only 1 in 3 sought help for these conditions in the preceding 12-month period [2]. In principle, the internet backed by a public health campaign might be a useful platform for reaching people with depression or anxiety who do not or are unable to access face-to-face health care. However, the use of the internet as a potential therapeutic platform raises a series of important concerns about data safety and privacy, effectiveness, user experience and adherence, exclusion of people without access to the internet, and data integration with care [3,4]. It is important to establish who might be reached by such an approach, whether they are already accessing mental health or general health services, and which recruitment approaches and interventions are most effective [5].

In this study, we explored the reach of a recruitment strategy for internet-based therapy directed at the public in one English county (Nottinghamshire, estimated population size of 914,000; aged >16 years in 2017, with 145,000 people with case-level depression and anxiety [6,7]). We used traditional and internet media and contacts through health, social care, and third-sector organizations for a randomized controlled trial (RCT) of 2 well-established digital services: the Big White Wall (BWW, now known as Togetherall) and Moodzone (MZ). BWW [8] offers (1) web-based assessment to assess common mental health problems and comorbid physical conditions; (2) moderated web-based peer support network: a community of peers, professionally staffed at all times, enabling safe support where individuals can choose if they wish to remain anonymous; and (3) guided support: a range of self-managed and facilitated programs for depression and anxiety based on cognitive behavioral therapy and social support principles. It was founded by a service user because existing services did not provide support when it was needed and was first developed as a collaboration among service users, digital experts, and a National Health Service (NHS) organization providing mental health services in London. Although BWW is widely available in many parts of England, there was little uptake of this intervention in Nottinghamshire before 2017. In England, the NHS provides its own free website with general information and contact details of local and national resources to help people with common mental health problems. It is known as NHS Mental Health and Well-being, previously called NHS Choices Moodzone [9].

To date, BWW and other web-based peer support for depression or anxiety have only been evaluated in RCTs conducted through primary or specialist mental health services rather than directly to the public [10-13]. There is uncertainty about how to conduct fully automated RCTs of digital mental health interventions directly targeted at the public [14]. In principle, fully automated RCTs without human contact are less prone to bias and can better elucidate actual treatment effects attributable to the digital intervention than those conducted with human conduct, where human contact may contribute to part of the treatment effect [15]. They can also be relatively inexpensive to run on a large scale [16]. However, it is unclear if and under what circumstances they would be feasible or acceptable to participants [17]. Automated trials have sometimes failed to engage some populations [18,19], whereas others offering otherwise difficult to obtain structured psychological treatments have been more successful [20-22].

### Objectives

This study has two main aims: (1) to investigate the reach, feasibility, and acceptability of a public health recruitment campaign using general media, digital media, health, third sector, and social services for a trial in people with probable mild-to-moderate depression or anxiety and (2) to test the feasibility, acceptability, baseline costs to society, and outcomes of conducting a fully automated RCT of 2 established digital interventions providing moderated web-based peer support and information (BWW) versus web-based information only (MZ).

## Methods

### Design

The Lived Experience Advisory Panel (LEAP) of patients and public representatives with personal experience of depression and anxiety that was formed for the study advised us on the best approaches to recruit people with depression and anxiety in the community. They recommended using the terms *low in mood* for depression and *stressed* for anxiety. We ran a public health campaign using these terms and offered the opportunity to take part in a study free of charge by comparing an information-giving website (MZ), which is a standard designed with and used within the NHS in the United Kingdom, with a web-based peer support site (BWW). We used a mix of traditional health research recruitment strategies, such as general practitioner (GP) endorsement, outpatient clinics, and support groups, as well as less traditional advertising, such as on buses and trams and via letter box leafleting. Special efforts were made to reach groups regarded as higher risk and harder to reach, such as the farming community. Reach was defined as the absolute number, proportion, and representativeness of individuals willing to participate in a given initiative [23].

The second stage of the study was a single-blind RCT using a fully automated bespoke study website. A member of the public could participate in the trial as they might do with other web-based applications, including consenting to the trial and all decision making, only seeking technical support if and when they need it. Full details are available in the published protocol [24]. Eligible participants self-referred and were recruited through the study website following a public health campaign. Consenting participants were randomly allocated to receive either 6 months of free access to BWW or signposted to the NHS MZ website.

Ethical approval was granted by the Local Research Ethics Committee (REC 16/EM/0204), and the final approval was received from the UK Health Research Authority.

### Public Health Campaign

The research team worked closely with a research delivery and support service (the National Institute of Health Research [NIHR] Clinical Research Network East Midlands), a professional marketing business (The Dairy), the study LEAP (18 people aged 25-65 years, 12 females), and the web developer (Ayup). The aim was to establish a brand for the study that was considered by the LEAP and study team to appeal to people who may be affected by low mood and/or stress in line with marketing materials but that would also instill professionalism and confidence with respect to the research project.

Trifold leaflets (Figure S1 in Multimedia Appendix 1), posters, bus and tram adverts, and business cards with quick response codes were subsequently developed, and a marketing plan for distribution and dissemination across Nottinghamshire was created for the duration of the study. We targeted tram routes across central Nottingham and bus routes that were purposefully targeted at the more deprived regions of the county. We took out newspaper and magazine advertisements and also spoke on local radio programs dedicated to health matters. We used a digital marketing agency (Nativve) to develop and implement a targeted local Facebook campaign and employed other social media, such as Twitter, to disseminate the study through appropriate networks.

In addition to the blanket approach that was adopted for the general public, the recruitment team also targeted particular groups, such as those in areas of greater deprivation, through door drops. Other targeted approaches involved asking organizations and health professionals to hand out leaflets or spread the word to their community to patients in their preferred method. For example, health visitors were asked to hand out leaflets to young parents, and Black and minority ethnic (BAME) groups were targeted through a third-sector organization, Awaaz. Places where the internet was accessed for free, such as self-help, third-sector groups, and local authority-funded libraries, were also asked to host leaflets and posters as well as to add these materials to their self-help sections.

Presentations were made by members of the research team to a range of GPs and primary care staff to raise awareness of the study across the county. These presentations were complemented by posters, cards, and leaflets that were displayed in every primary care waiting room in Nottinghamshire. Community pharmacists were targeted through their health promotion work and asked to promote the study through displaying the leaflets and posters, and when they considered it appropriate, verbally informing people who might be collecting medication for their mental health that a trial was being conducted. We targeted other health and social care workers, including educational establishments, such as universities, further education colleges, and those in contact with socially isolated groups, for example, social workers, health visitors, or those who work with mental health problems such as the Improving Access to Psychological Treatment program providing psychological treatments for depression and anxiety, private counselors and wellness in mind, and an NHS-funded public mental health signposting service.

### Sample and Eligibility

Potential participants from the county of Nottinghamshire self-referred to the study, and their eligibility was assessed by an automated digital program on the study website. The study website requested the GP’s contact details when the person was ineligible for the study.

Inclusion criteria were as follows: patients (1) aged ≥16 years; (2) reside in the county of Nottinghamshire; (3) scored between 10 and 20 on the 9-item Patient Health Questionnaire (PHQ-9) [25] or 10 or more on the 7-item Generalized Anxiety Disorder questionnaire (GAD-7) [26], indicating probable caseness for depression and anxiety, respectively, but not a definite diagnosis of depression or anxiety disorder; (4) had access to the internet through a computer, tablet, or smartphone (Windows, iOS, or Android) device and email address; and (5) were able and willing to give informed consent (through electronic consent).

Exclusion criteria were as follows: patients (1) scored ≥21 on the PHQ-9 (severe depression); (2) scored 2 or 3 on PHQ-9 item “thoughts that you would be better off dead or of hurting yourself in some way”; and (3) scored ≤10 on PHQ-9 and GAD-7.

BWW and MZ are only available in the English language. Therefore, the website recommended nonparticipation for those who believed they were insufficiently proficient in the use of the English language. There was no test of proficiency in English or information technology literacy.

Participants were ineligible for the trial because they scored in the severe range on the PHQ-9 or scored 2 or 3 on the suicide item of the PHQ-9. In line with other digital studies [27], a national research committee did not allow us to recruit these potential participants because, in their opinion, research into people with severe depression requires a greater duty of care than could be offered over the internet. These excluded participants were provided with an opportunity to request that the study team inform their GP, mental health care team, or caregiver of their current mood state. If the request was not completed, the study team followed up via email, asking if they would like the team to inform their GP or care team. We followed up the excluded participants on one occasion.

Information on the participants and the associated consent forms were provided electronically within the study website. Participants who wished to discuss the study could email and telephone the study team if they had any further questions before consenting to the study. An email confirming consent was sent to each participant once they had fully enrolled.

### Interventions

#### Randomization: Arm 1—BWW

Participants allocated to receive 6 months of free access to the BWW website [8] were invited to create a user profile using a pseudonym that was linked to the trial identification to which they had been assigned within the study website. They had to create a profile within 14 days of being randomized. Participants were able to access any part of the BWW site (apart from the option of personalized therapy or counseling sessions that have to be prescribed by a clinician, that is, not offered directly to the general public) and interact with other users within the boundaries of the site’s house rules. Anonymized records of log-ins, time on site, interactions, and page categories were recorded by BWW on behalf of the study team.

#### Randomization: Arm 2—Participants Allocated to MZ

Participants were directed to the MZ area of the NHS Choices website [9]. Participants were able to access all available materials on mental health, including depression and anxiety. We did not have records of time on site or use of the site. NHS MZ access was used as the control digital resource, so all participants were offered some help for their problems with depression or anxiety, but this control group did not have access to moderated, anonymized peer social support.

### Outcome Measures

Once consented, participants were asked to complete self-rated questionnaires to measure well-being, depression, anxiety, work and social adjustment, receipt of services (for economic analysis), social support, and personality dysfunction at baseline. These were completed on the web (though the study website) for approximately 20 to 30 minutes. All data were stored on the website and downloaded and anonymized by the clinical trial manager.

Participation in the study lasted for 6 months. Participants received electronic follow-up invitations at 3, 6, 12, and 26 weeks after randomization to be completed on the website. Each participant was reminded to log onto the study website and complete follow-up measures by email 24 hours before each follow-up and at the follow-up time point. If follow-up was not completed, they received another reminder 48 hours later. Participants were emailed motivational statements encouraging follow-up as well as the offer of entry into a prize draw at the end of the study if they completed at least the primary outcome measure in all follow-up assessments. There were no other attempts to follow up participants using any form of digital, telephone, or face-to-face contact.

#### Primary Outcome Measure

The primary outcome measure is change in self-rated well-being from baseline to 6 weeks after baseline using the 14-item Warwick-Edinburgh Mental Well-being Scale (WEMWBS) [28].

#### Secondary Outcomes Measures

The secondary outcome measures are as follows:

- Well-being was measured at 3, 12, and 26 weeks using the WEMWBS.
- The GAD-7 [25] was completed as part of eligibility at baseline and at 3, 6, 12, and 26 weeks, as a measure of anxiety severity.
- The PHQ-9 [26] was completed as part of eligibility at baseline and at 3, 6, 12, and 26 weeks, as a measure of depression severity.
- Social function on the 8-item Work and Social Adjustment Scale [29], a measure of function, was completed at baseline and at 3, 6, 12, and 26 weeks.

#### Baseline Measures

At baseline, basic sociodemographic characteristics were collected along with measures of health and social care resource use over the 3 months before study entry [30], social support [31], life events over the previous 3 months [32], and personality dysfunction [33].

### Modifications to the Original Protocol Conducted in Real Time Based on Participant Feedback

Feedback left by the first 50 participants suggested that they disliked the intrusiveness and length of some of the measures and assessments at baseline. One participant withdrew from the study for this reason. Therefore, compared with our protocol [24], we omitted the 12-item medical outcomes study short-form health survey version 2.0 (SF-12) [34] at all time points and only carried out the economic resource proforma [31] at baseline. At baseline, the number of questions asked fell from 92 to 80 and at each follow-up time point from 50 to 38.

### Sample Size

The sample size calculation and justification is outlined in detail in our protocol paper [24]. A total of 676 patients were needed to detect a 3-point (SD 12), minimal clinically important difference for adults on the 14-item WEMWBS [35] at a significance level of .05 with 90% power. After adjusting for a 50% attrition rate at 6 weeks [36], a total of 1352 participants were required for our RCT.

### Randomization and Monitoring

The treatment to which a participant was assigned was determined by a computer-generated pseudorandom code using random permuted blocks of varying sizes by a randomization system embedded within the website. No stratification or minimization was performed. Treatment assignment was relayed by the computer program to the participant and opened to the trial manager (CK) who monitored recruitment, data completion, and technical problems with the website.

### Statistical Analysis

Feasibility and acceptability was assessed by recruitment and retention during follow-up using descriptive statistics. All analyses were performed on an intention-to-treat basis by a trial statistician blinded to treatment allocation using STATA 16 (StataCorp LLC). As all outcome scores were repeatedly measured at baseline and at 3, 6, 12, and 26 weeks, multilevel modeling was performed to quantify the treatment effect with participants as a level 2 unit and baseline, treatment arm, follow-up time, and interaction of arm×time as a covariate. Missing outcome values were investigated and imputed for all outcomes under the missing at random assumption with 100 data sets imputed for data analysis. REALCOME and STATA 16 were used to impute missingness. Similar models were conducted on observed values to check the robustness of the treatment effect estimates sensitive to the influence of missingness.

### Health Economics

We electronically administered the Client Service Receipt Inventory [30] to participants at baseline, which collected data on NHS service use and other costing variables [37-44]. Owing to the inherent comorbidity of mental and physical health, these questions pertained to all service use and all health-related absenteeism in place of condition-attributable service use, as we wished to capture possible changes in service use and service-seeking behaviors. The withdrawal of the SF-12 instrument meant that we were unable to examine the health state utilities associated with mild-to-moderate depressive episodes and anxiety disorders.

The costs of health-related time taken off work (absenteeism) were estimated using the lost wages approach [45]. We adopted a median team multiplier, as although wages are a suitable estimate of marginal productivity losses to businesses, this measure tends to be an underestimation for individuals working in team environments [46]. We assigned population-level gross weekly salaries to individuals by full-time or part-time employment status and gender. We designated participants as part time if they were employed or self-employed and worked less than 30 hours per week and as full time if they were employed or self-employed and worked for more than 30 hours. We note that this does not attribute any value to health-related presenteeism or those who are unemployed owing to ill health.

The prevalence of mild-to-moderate depression and anxiety was derived using the Adult Psychiatric Morbidity Survey [42] by combining the severity of symptoms of common mental disorders, where a score between 12 and 17 represents a diagnosable mild-to-moderate condition, with common mental disorder in the past week using the Clinical Interview Schedule Revised score. We extrapolated productivity losses using disease prevalence by gender to control for observed self-selection and the number of individuals between 18 and 64 years of age active in the labor market (employed or self-employed) in the United Kingdom. Our estimates of direct-to-NHS costs used a larger population, including those who are inactive in the labor market. The data sources of NHS unit costs and the resources used to estimate productivity losses are displayed in Multimedia Appendix 2 (Table S1).

To control for missingness, we assumed the item response was missing at random. Multiple imputation was run following best practices [47,48] for a total of 50 imputed sets (m50) [49]. The final model specification included variables for gender, WEMWBS, PHQ-9, age, education, and employment status alongside our outcomes of interest. Multiple imputation was inclusive of both trial arms to increase the sample size because neither arm had received treatment at baseline. Models for direct-to-NHS costs and productivity losses were conducted separately, at the item response level, because of the inherent subsampling of productivity losses to only those in employment. Models were run multiple times, and distributions were visually inspected to confirm the robustness and stability of our imputations. The 95% bias-corrected CIs were derived from 1000-iteration bootstraps, and all health economics analyses were conducted in Stata/SE 16.1. Pounds sterling were converted to US dollars using the conversion rate published by the Federal Reserve as of March 26, 2021: £1 to US $1.38.

### Data on Barriers and Facilitators

We asked for open-ended survey feedback at baseline and every follow-up and also received email and telephone feedback when participants wished to contact the research team. We then analyzed this feedback thematically.

## Results

### Overview

**Figure 1:**
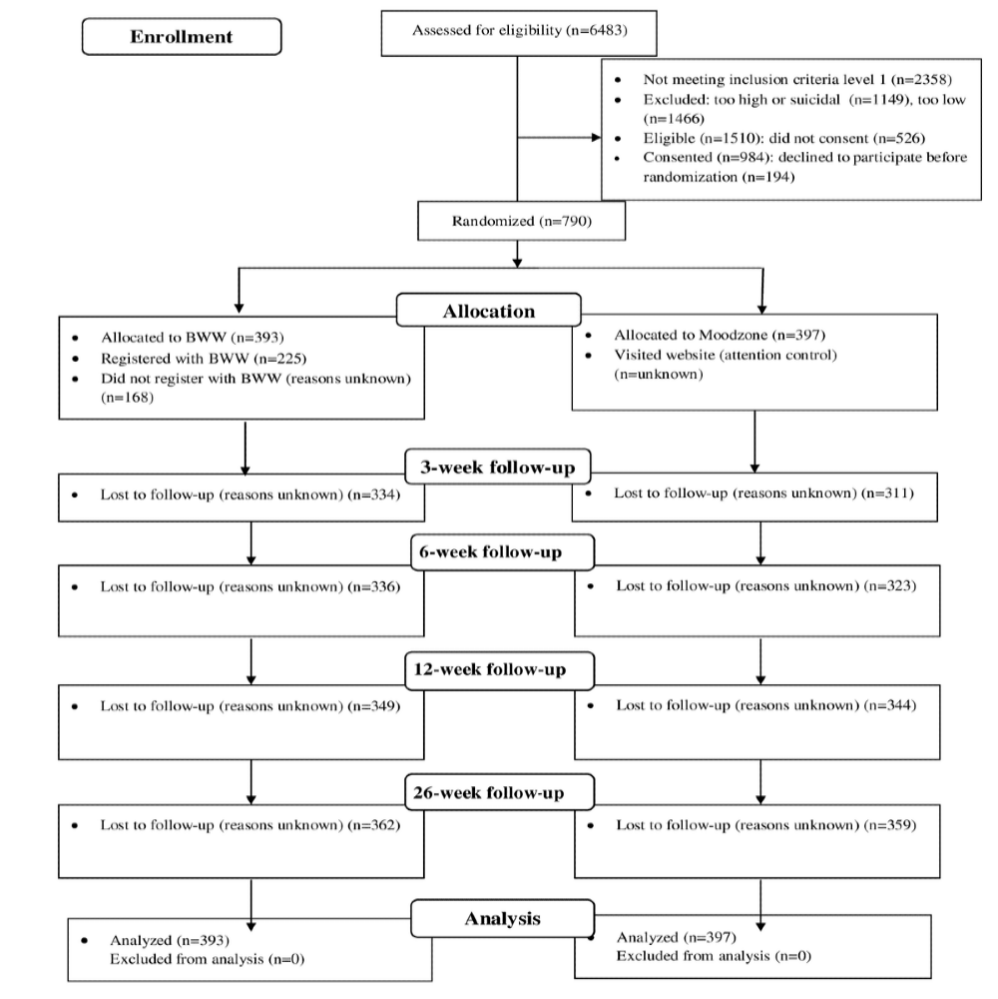
Flow of participants through the study.

Figure 1 shows that there were 6483 visitors to the study website (14 per day) over 18 months of recruitment from September 16, 2016, to May 30, 2018. Of these, 4125 were from Nottinghamshire, aged >16 years, and had continued access to the internet. We excluded a further 1149 participants because their PHQ-9 scores were above 20 or because they scored 2 or 3 on the suicide item of the PHQ-9 and excluded 1466 participants who scored below 10 on both the PHQ-9 and GAD-7. Of the 1510 eligible participants, 790 consented and were randomized, 393 to MZ and 397 to BWW.

### Methods of Recruitment

Table 1 shows that the 6 most successful ways of recruiting visits to the website were through a university counseling service, direct contact with the research team at presentations, leaflets, pharmacies, and health visitors. However, the 6 most successful enrollment methods to the study (randomization) were through GPs, Facebook, trams, internet and social media, NHS acute hospitals, and leaflet door drop. In relation to primary care, 22,408 registered patients received a text message from their GP endorsing the study, and 180 people were consented and randomized into the study via this route. The efforts directed at raising awareness of this study are listed in Table S2 (Multimedia Appendix 2). Figures S2 and S3 (Multimedia Appendix 2) show recruitment by the geography of Nottinghamshire and in relation to the proximity of tram routes in the county, suggesting that proximity to tram routes in the City of Nottingham was associated with study enrollment.

**Table 1:** Number of people visiting the study website and those converted to randomized study participants according to their self-reported referral source.

Source	People visiting the study website (N=6483)^a^, n (%)	People randomized (percent converted by a referral source; n=790)^a^, n (%)	Randomized population by referral source (n=790), n (%)
Awaaz (charity)	2 (0.03)	0 (0.0)	0 (0.0)
Bus advertisement	65 (1.00)	8 (12.3)	8 (1.0)
Direct contact	9 (0.14)	4 (44.4)	4 (0.5)
Door drop	250 (3.87)	58 (23.2)	58 (7.3)
Education	62 (0.96)	13 (20.9)	13 (1.7)
Employer	109 (1.68)	12 (11.0)	12 (1.5)
Facebook	1050 (16.20)	136 (12.9)	136 (17.2)
Family	17 (0.26)	3 (17.7)	3 (0.4)
Friend	213 (3.29)	36 (16.9)	36 (4.6)
General practitioner	977 (15.07)	180 (18.4)	180 (22.8)
Health visitor	15 (0.23)	4 (26.7)	4 (0.5)
Library	30 (0.46)	5 (16.7)	5 (0.6)
Leaflet	48 (0.74)	15 (31.3)	15 (1.9)
Internet	751 (11.58)	63 (8.4)	63 (7.9)
Improving access to psychological therapies program	49 (0.76)	10 (20.4)	10 (1.3)
Magazine	18 (0.28)	1 (5.6)	1 (0.1)
MIND	12 (0.19)	1 (8.3)	1 (0.1)
Newspaper	10 (0.15)	1 (10.0)	1 (0.1)
National Health Service	334 (5.15)	56 (16.8)	56 (7.1)
No recall	1 (0.02)	0 (0.0)	0 (0.0)
Other (unknown)	34 (0.52)	5 (14.7)	5 (0.6)
Pharmacy	135 (2.08)	42 (31.1)	42 (5.3)
Poster	9 (0.14)	2 (22.2)	2 (0.3)
Radio	47 (0.72)	9 (19.2)	9 (1.1)
Social worker	5 (0.08)	0 (0.0)	0 (0.0)
Support service	44 (0.68)	10 (22.7)	10 (1.3)
Tram advertisement	371 (5.72)	67 (18.1)	67 (8.5)
Twitter	20 (0.31)	1 (5.0)	1 (0.1)
University counseling service	17 (0.26)	8 (47.1)	8 (1.0)
Voluntary (third-sector) group	10 (0.15)	2 (20.0)	2 (0.3)
Wellness in mind	5 (0.08)	1 (20.0)	1 (0.1)
No response to question	1764 (27.21)	38 (2.2)	38 (4.8)

^a^Participants were asked how they heard about the study.

### Trial Results

Table 2 shows that randomized study participants had a mean age of 38.0 (SD 13.8) years, 81.0% (640/790) were female, 93.4% (738/790) were White, everyone had educational qualifications with 45.0% (354/787) having a university degree, 4.3% (34/790) were retired, and 8.9% (70/790) were unemployed. The mean scores showed moderate levels of depression and anxiety and moderate impairments in function and well-being. Of the 393 participants randomized to BWW, 225 (57.3%) registered to access BWW and 165 (42.5%) accessed it on more than one occasion. No participation data are available for the MZ website.

**Table 2:** Demographic and clinical characteristics of study participants (n=790).

Characteristics		Randomized to Moodzone (n=397)^a^	Randomized to Big White Wall (n=393)^a^
Age (years), mean (SD)		38.4 (14.3)^b^	37.6 (13.7)^c^
Females, n (%)		316 (79.6)	324 (82.4)
**Ethnicity group, n (%)**			
	White	370 (93.2)	368 (93.6)
	South Asian	7 (1.8)	8 (2.0)
	Black	5 (1.3)	3 (0.7)
	Other	11 (2.8)	14 (3.6)
	Missing	4 (1.0)	0 (0.0)
**Highest educational attainment, n (%)**			
	Degree or higher (higher education)	175 (44.1)	179 (45.5)
	A levels or Business and Technology Education Council (further education)	126 (31.7)	118 (30.0)
	General Certificate of Secondary Education or National Vocational Qualification (basic secondary school)	93 (23.4)	96 (24.4)
	No qualifications	0 (0.0)	0 (0.0)
	Missing	3 (0.8)	0 (0.0)
**Employment, n (%)**			
	Employed	254 (64.0)	249 (63.0)
	Student or training	65 (16.4)	79 (20.1)
	Retired	20 (5.0)	14 (3.6)
	Unemployed	41 (10.3)	29 (7.4)
	Other	17 (4.3)	22 (5.8)
**Well-being and mental health assessment scores at study enrollment (self-administered by participant), mean (SD)**			
	Warwick-Edinburgh Mental Well-Being Scale	34.54 (5.72)^d^	34.54 (5.56)^e^
	7-item Generalized Anxiety Disorder Questionnaire	12.99 (3.13)	12.76 (2.91)
	9-Item Personal Health Questionnaire	13.66 (2.55)	13.61 (2.54)
	Work and Social Adjustment Scale	22.93 (7.07)^f^	23.50 (6.94)^g^

^a^Number of individuals reported only when characteristics were not reported by all participants.

^b^n=309.

^c^n=304.

^d^n=372.

^e^n=359.

^f^n=359.

^g^n=336.

Of the 790 participants enrolled in the study, 572 (72.4%) provided complete case (CC) data on health care service use at baseline. The discussed results correspond to the reported participant service use in the preceding 3 months to baseline (Table 3). Of the 572 patients, no contact with any health service was reported by 271 (47.4%) patients of the CC sample. Out of the 572 patients, only 66 (11.5%) had any contact with mental health services, 228 (39.9%) had contact with their GP and 9 (1.6%) had out-of-hours care. Multimedia Appendix 2 (Tables S1 and S3) shows unit costs, productivity resources, disease prevalence, and CCs of intensity of service use.

**Table 3:** Baseline self-reported service use in the 3 months before study entry.

Service^a^		Randomized to Moodzone (n=397)		Randomized to Big White Wall (n=393)		Total (N=790)	
		Total, n	Patients, n (%)	Total, n	Patients, n (%)	Total, n	Patients, n (%)
Any service use		293	155 (52.9)	279	146 (52.3)	572	301 (52.6)
**Inpatient**		269	23 (8.6)	267	15 (5.6)	536	38 (7.1)
	General medical ward	257	7 (2.7)	279	8 (2.9)	536	15 (2.8)
	Acute psychiatric ward	257	2 (0.8)	279	0 (0.0)	536	2 (0.4)
**Outpatient**		282	82 (29.1)	277	70 (25.3)	559	152 (27.2)
	Emergency room	273	20 (7.3)	286	18 (6.3)	559	38 (6.8)
	Radiology	273	17 (6.2)	286	19 (6.6)	559	36 (6.4)
	Physiotherapist	273	17 (6.2)	286	13 (4.5)	559	30 (5.4)
	Occupational therapist	273	6 (2.0)	286	6 (2.1)	559	12 (4.6)
	Psychiatrist	273	15 (5.5)	286	13 (4.5)	559	28 (5.0)
**Primary and community**		292	115 (39.4)	279	119 (42.7)	571	234 (41.0)
	GP^b^	291	113 (38.8)	279	115 (41.2)	570	228 (40.0)
	GP home visit	291	2 (0.7)	279	2 (0.7)	570	4 (0.7)
	Practice nurse	291	31 (11.0)	279	38 (13.6)	570	69 (12.1)
	Psychologist	291	6 (2.1)	279	3 (1.1)	570	9 (1.6)
	Psychiatric Nurse	291	4 (1.4)	279	5 (1.8)	570	9 (1.6)
	Occupational Therapist	291	2 (0.7)	279	6 (2.2)	570	8 (1.4)
	Out-of-hours care	291	4 (1.4)	279	5 (1.8)	570	9 (1.6)
	Walk-in center	291	5 (1.7)	279	10 (3.6)	570	15 (2.6)
	Social worker	291	1 (0.3)	279	2 (0.7)	570	3 (0.5)
	Private counseling or therapy	293	11 (3.8)	276	7 (2.5)	569	18 (3.2)
	Other use	293	3 (1.0)	276	9 (3.3)	569	12 (2.1)
	No reported information on service use	397	104 (26.2)	393	114 (29.0)	790	218 (27.6)

^a^Other use consisted of other specific National Health Service care, that is, phlebotomist or private health care, that is, physiotherapy and podiatry. Designations of the community psychiatrist were included as outpatient psychiatrist for the 4 participants who double counted by listing psychiatrists within other service use. Binary variables for inpatient or outpatient or primary and community aggregate service use were amended to 1 or 0 if missing and participants specified individual service contact or no individual service use, respectively. If binary variables declared no aggregate service use, individual service use binary variables were set to 0 if missing, and no other individual service use was observed.

^b^GP: general practitioner.

We report the direct-to-NHS costs and productivity losses in Table 4. Participants in employment took a mean 10.93 (95% CI 9.51-12.36) days of health-related time off work during the 3 months, which corresponded to a productivity loss of £1001.01 (95% CI 868.75-1133.27; US $1380.79, 95% CI 1198.35-1563.23), compared with £156.46 (95% CI 114.08-198.84; US $215.82, 95% CI 157.36-274.28) in direct-to-NHS costs per participant. The small variation between the CC and multiply imputed costs suggests that the observed characteristics are poor predictors of response missingness.

**Table 4:** Direct-to-National Health Service costs and productivity losses in the 3 months before study entry.

Variable		Population, n (%)	Mean (95% CI)	Total annual burden, £ (US $)
**Complete case**				
	Absentee days^a^ (n=494)	257 (52)	11.01 (8.62-13.80)	N/A^b^
	Productivity losses (n=494)	257 (52)	£995.74 (775.27-1251.21; US $1373.52 [1069.41-1725.92])	2,336,113,572 (3,222,435,061)
	Direct-to-NHS^c^ costs (n=790)	483 (61)	£158.75 (119.23-209.13; US $218.98 [164.47-288.47])	505,479,259 (697,258,089)
**Multiple imputation**				
	Absentee days (n=494)	494 (100)	10.93 (9.51-12.36)	N/A
	Productivity losses (n=494)	494 (100)	£1001.01 (868.75-1133.27; US $1380.79 [1198.35-1563.23])	2,348,477,561 (3,239,489,947)
	Direct-to-NHS costs (n=790)	790 (100)	£156.46 (114.08-198.84; US$ 215.82 [157.36-274.28])	498,187,621 (687,200,004)

^a^Days taken off work because of ill health of those employed or self-employed.

^b^N/A: not applicable.

^c^NHS: National Health Service.

Table 5 shows that follow-up assessment rates were very low at each time point. At baseline, 93.5% (739/790) participants completed the WEMWBS, but only 18.4% (145/790) completed it at 3 weeks, 16.6% (131/790) completed it at 6 weeks (the primary outcome), 12.3% (97/790) completed it at 12 weeks, and 8.7% (69/790) completed it at 26 weeks. Proportions of participants completing the primary outcome measure were 14.5% (57/393) in the BWW intervention group and 18.6% (74/397) in the MZ control group. There were no statistically significant differences in the primary outcome WEMWBS at 6 weeks between the 2 treatment groups nor were there any differences at other time points or in the PHQ-9, GAD-7, or Work and Social Adjustment Scale in the imputed or observed results.

**Table 5:** Modeled mean changes in well-being and mental health scores (95% CIs) at each study follow-up time.

Outcome and follow-up time		Randomized to Moodzone (n=397)			Randomized to Big White Wall (n=393)			Difference	
		Mean (95% CI)	Participant, n (%)^a^	Mean (95% CI)		Participant, n (%)^a^	Mean (95% CI)		P value
**7-item Generalized Anxiety Disorder scale**									
	3 weeks	−2.37 (−3.29 to −1.45)	87 (21.9)	−1.91 (−3.08 to −0.74)		57 (14.3)	0.46 (−1.04 to 1.96)		.55
	6 weeks	−2.53 (−3.53 to −1.52)	74 (18.6)	−2.13 (−3.21 to −1.05)		55 (14.0)	0.40 (−1.24 to 2.03)		.63
	12 weeks	−2.51 (−3.67 to −1.35)	53 (13.4)	−3.02 (−4.23 to −1.81)		44 (11.2)	−0.51 (−2.26 to 1.24)		.57
	26 weeks	−3.26 (−4.63 to −1.89)	38 (9.6)	−3.62 (−5.12 to −2.12)		31 (7.9)	−0.36 (−2.32 to 1.60)		.72
**9-item Patient Health Questionnaire**									
	3 weeks	−1.83 (−2.85 to −0.81)	84 (21.1)	−1.39 (−2.69 to −0.09)		58 (14.8)	0.44 (−1.11 to 1.98)		.58
	6 weeks	−1.92 (−2.97 to −0.87)	74 (18.6)	−1.19 (−2.55 to 0.17)		56 (14.2)	0.73 (−0.88 to 2.35)		.37
	12 weeks	−2.07 (−3.29 to −0.86)	53 (13.4)	−2.25 (−3.65 to −0.84)		44 (11.2)	−0.17 (−1.92 to 1.58)		.84
	26 weeks	−3.36 (−4.62 to −2.10)	38 (9.6)	−2.96 (−4.62 to −1.30)		30 (7.6)	0.40 (−1.69 to 2.49)		.70
**Work and Social Adjustment Scale**									
	3 weeks	−1.31 (−2.97 to 0.35)	79 (19.9)	−2.85 (−5.07 to −0.62)		54 (13.7)	−1.54 (−4.47 to 1.40)		.30
	6 weeks	−1.06 (−2.94 to 0.82)	71 (17.9)	−2.68 (−4.83 to −0.52)		51 (13.0)	−1.62 (−4.58 to 1.34)		.28
	12 weeks	−1.66 (−3.58 to 0.26)	52 (13.1)	−3.24 (−5.74 to −0.73)		39 (9.9)	−1.57 (−5.07 to 1.92)		.37
	26 weeks	−4.21 (−6.39 to −2.02)	37 (9.3)	−5.95 (−8.57 to −3.33)		29 (7.4)	−1.74 (−5.08 to 1.59)		.30
**Warwick-Edinburgh Mental Well-being Scale**									
	3 weeks	2.69 (1.08 to 4.29)	86 (21.7)	2.56 (0.80 to 4.33)		59 (5.0)	−0.12 (−2.55 to 2.30)		.92
	6 weeks^b^	3.15 (1.42 to 4.89)	74 (18.6)	1.96 (−0.06 to 3.99)		57 (14.0)	−1.19 (−3.71 to 1.33)		.35
	12 weeks	3.92 (1.86 to 5.98)	53 (13.4)	4.29 (2.08 to 6.49)		44 (11.2)	0.37 (−2.64 to 3.38)		.81
	26 weeks	5.35 (3.47 to 7.23)	38 (9.6)	6.63 (4.25 to 9.02)		31 (7.4)	1.29 (−1.64 to 4.21)		.39

^a^Participants with available scores at each time point.

^b^Primary outcome of randomized controlled trial.

### Barriers to Participation and Retention in the Study

We collected the following feedback organized into 3 themes on barriers to participation and retention in the study from randomized participants, those who had considered participating, and those who refused to participate but left comments for the research team by email, text, or survey.

#### Lack of Personal Interaction With the Research Team

The study was set up to be automated so that a member of the public could participate in the trial, as they might do with other web-based applications, only seeking technical support if and when they needed it. However, this meant a lack of personal connection and engagement with the research team. Participants described this as contributing to a lack of obligation to complete the study measures and participate in follow-up time points. They viewed the interventions as similar information-giving interventions that were impersonal, with many participants not understanding how the interventions might be tailored to their needs.

#### Turning Away People With Severe Depression

Potential participants with more severe depression who were trying to take part but were turned away by the automated eligibility criteria on the REBOOT website expressed disappointment, frustration, and a sense of exclusion made apparent through a number of complaints to the study’s email account.

#### Lack of Technical Support

Although people contacted the research team over technical problems, contact with the research team usually led to greater engagement by those participants during follow-up. The study experienced technical issues, such as website downtime, problems with progression through the site, and errors within the measures, which may have deterred the completion of some measures and retention in the study.

## Discussion

### Principal Findings

Overall, we found that running a fully automated, web-based intervention trial was challenging. Exclusion criteria were exclusive web-based enrollment and measurement, restricted full enrollment, and retention during follow-up. We recruited and randomized only 790 of 1510 (52.3%) of those who expressed interest and were eligible, despite a considerable amount of effort by the research team using traditional advertising, internet and social media, health services, other public services, and third-sector contacts. Only 16.6% (131/790) of the patients completed the primary outcome at 6 weeks. As a result, the trial lacked the power to demonstrate any differences in outcome between BWW and MZ. The primary reasons for the lack of recruitment and poor retention in this automated RCT are discussed.

There was a demand for web-based information and peer support for the sort offered by the study intervention from people with severe depression and those who were actively suicidal. Far more of these visitors to the website were willing to participate in the study (1149/4125, 27.85%) than those with mild-to-moderate depression and anxiety who were enrolled (790/4125, 19.15%). We were not allowed to recruit these participants by a research ethics committee because of the opinion that research on people with severe depression requires a greater duty of care than could be offered over the internet. However, such patients access digital services on an everyday basis outside the research environment. A restrictive approach to research ethics may have an undesirable effect in that research into digital interventions is not carried out in the most vulnerable manner, where there is arguably more room for beneficial effects and greater safety concerns [50,51].

Participants were most successfully enrolled through GPs (directly and through texting from the practice), pharmacies, internet resources such as Facebook and social media, public transport advertising, and door-drop–posted information in more deprived communities. We recruited a largely female, White, and educated sample who were mostly currently in work or education. Approximately half of those enrolled were not in contact with any health service in the preceding 3 months. A core aim of providing digital mental health approaches to reach people who are not in contact with health services was achieved. Younger females are a part of the population with increasing rates of depression and anxiety in the United Kingdom [2], suggesting that such people might be reached through digital direct-to-public services. However, we failed to recruit enough males, older participants, people from BAME backgrounds, people without any educational qualifications, and people in more rural areas, each of whom may require a combination of different strategies for enrollment.

Compared with the UK average of 4.1 sickness absence days of the general working population, our employed participants took a mean of 43.72 (95% CI 38.04-49.44) days off work per annum, with many individuals undergoing prolonged absences of illness from work. Individuals with mild-to-moderate depression or anxiety may represent a conservative annual burden of approximately £498 million (US $687 million) to the NHS and an additional £1.42 billion (US $1.96 billion) in productivity losses. Our extrapolation to a 1-year time horizon may underestimate or overestimate this burden. There exists a significant value for treating these individuals outside of the standard measures of health gain.

From feedback to the study, the decision to take part in the study was made very quickly by participants, many of whom were not necessarily committed to completing it. Participants found all contact with the study remote and therefore not engaging. Our research group has published a systematic review and meta-synthesis of qualitative data on enrollment and retention in the study or treatment from 24 trials of digital interventions with varying degrees of human engagement in enrollment, follow-up, or treatment of people with depression and anxiety. It identified that enrollment and retention to studies were determined by the participants’ initial beliefs about digital health interventions, the offer of personal support, and the enablement of personalization of care [52]. Taking our results together with this meta-synthesis, the public health campaign and automated enrollment only provided a superficial understanding of what was being offered by the trial. The opportunities provided by BWW and MZ to use novel approaches to making choices about obtaining personal support and personalized care, particularly through BWW, which is designed to enable such choices to be made and provided almost immediately, were not made explicit and might have been explained better through initial human or virtual human contact. Those trials that have been automated and yet recruited and retained a high proportion of participants offered well-established treatments for depression and anxiety (cognitive behavioral therapy) to populations that could not otherwise access such help [20-22]. The concept of building a program of tailored personal support and formal psychoeducation through interaction with peers and trained guides that BWW offers was not well-known in the county of Nottinghamshire at this time. Better recruitment and retention rates were obtained in another trial of BWW in mental health service users involving more human support [10]. Therefore, our data suggest that a process of interaction with a human or possibly a virtual human is required to ensure a full understanding of what the trial offered, fully informed consent, and commitment to the study, particularly when the digital interventions are not already well understood in the population being studied.

Participants commented on how burdensome they had found the length of the questionnaires, and longer assessments in digital studies may deter participation [53]. Therefore, during the recruitment of the first 50 participants, we decided to stop collecting questionnaires, namely the SF-12. However, this change did not improve either recruitment or retention in the study.

In this study, participants were routed to 2 other websites, BWW and MZ, which each had the option of completing questionnaires examining their mental state similar to our follow-up measures. These may have confused or deterred participants from completing follow-up measures in the study. We only used email reminders of follow-up and did not use reminders through social media, telephone, or arranged face-to-face contact. More persistent follow-up using a variety of different methods might have improved follow-up rates and provided greater clarity that mental state questionnaires on BWW were independent of follow-up questionnaires in the study. We employed motivational statements and the opportunity to take part in a raffle to win store vouchers at the end of the study. Although these potential intrinsic and extrinsic rewards may sometimes improve retention and follow-up assessment rates in RCTs [54], they are insufficient.

### Conclusions

This study shows that the offer directed to the public of a trial of peer support and information-providing interventions for people with probable mild-to-moderate depression and/or anxiety backed by a public campaign was successful in reaching some parts of the population not in contact with primary care or secondary care mental health services. Most of these were White, educated women who were working and studying below the age of 50 years, who were costly in terms of loss of productivity. However, the public health campaign was not successful in enrolling a large number of high-risk groups for depression or anxiety: men, BAME communities, older people, poorly educated people, and people living in rural communities with poor access to traditional services. This fully automated trial was not successful in engaging or retaining participants because it did not recruit people with severe depression who most wanted these interventions and did not adequately explain how the digital interventions could provide personalized care and support.

## Appendix

Multimedia Appendix 1 Trifold leaflet.

Multimedia Appendix 2 Further recruitment and economic data.

Multimedia Appendix 3 CONSORT-eHEALTH checklist (V 1.6.1).

## Abbreviations

BAME: Black and minority ethnic
BWW: Big White Wall
CC: complete case
GAD-7: 7-item Generalized Anxiety Disorder
GP: general practitioner
LEAP: Lived Experience Advisory Panel
MZ: Moodzone
NHS: National Health Service
NIHR: National Institute of Health Research
PHQ-9: 9-item Patient Health Questionnaire
RCT: randomized controlled trial
SF-12: 12-item medical outcomes study short-form health survey
WEMWBS: Warwick-Edinburgh Mental Well-being Scale
