# Correction: A Direct-to-Public Peer Support Program (Big White Wall) Versus Web-Based Information to Aid the Self-management of Depression and Anxiety: Results and Challenges of an Automated Randomized Controlled Trial

**DOI:** 10.2196/31543

**Published:** 2021-07-13

**Authors:** Richard Morriss, Catherine Kaylor-Hughes, Matthew Rawsthorne, Neil Coulson, Sandra Simpson, Boliang Guo, Marilyn James, James Lathe, Paul Moran, Laila J Tata, Laura Williams

**Affiliations:** 1 Institute of Mental Health University of Nottingham Nottingham United Kingdom; 2 Department of General Practice University of Melbourne Melbourne United Kingdom; 3 School of Medicine University of Nottingham Nottingham United Kingdom; 4 Research Delivery Team Nottinghamshire Healthcare NHS Foundation Trust Nottingham United Kingdom; 5 Centre for Longitudinal Studies University College London London United Kingdom; 6 School of Medicine University of Bristol Bristol United Kingdom

In “A Direct-to-Public Peer Support Program (Big White Wall) Versus Web-Based Information to Aid the Self-management of Depression and Anxiety: Results and Challenges of an Automated Randomized Controlled Trial” (J Med Internet Res 2021;23(4):e23487), one error was noted.

Due to a system error, the name of one author, Laila J Tata, was replaced with the name of another author on the paper, Laura Williams. In the originally published paper, the order of authors was listed as follows:

Richard Morriss, Catherine Kaylor-Hughes, Matthew Rawsthorne, Neil Coulson, Sandra Simpson, Boliang Guo, Marilyn James, James Lathe, Paul Moran, Laura Williams, Laura Williams

This has been corrected to:

Richard Morriss, Catherine Kaylor-Hughes, Matthew Rawsthorne, Neil Coulson, Sandra Simpson, Boliang Guo, Marilyn James, James Lathe, Paul Moran, Laila J Tata, Laura Williams

In the originally published paper, the ORCID of author Laura Williams was incorrectly published as follows:

0000-0002-6404-8658

This has been corrected to:

0000-0001-6296-6677

The correction will appear in the online version of the paper on the JMIR Publications website on July 12, 2021, together with the publication of this correction notice. Because this was made after submission to PubMed, PubMed Central, and other full-text repositories, the corrected article has also been resubmitted to those repositories.

