# Supplementary material for: A Direct-to-Public Peer Support Program (Big White Wall) Versus Web-Based Information to Aid the Self-management of Depression and Anxiety: Results and Challenges of an Automated Randomized Controlled Trial
Source: J Med Internet Res. 2021 Apr 23;23(4):e23487. doi: 10.2196/23487 (PMC8105759; doi:10.2196/23487)
Supplement: Multimedia Appendix 1 [file jmir_v23i4e23487_app1.pdf]

About the Study

REBOOT is a study set up to see whether an online peer support website **Big White Wall** is more or less effective in helping people if they are experiencing symptoms of depression and anxiety, compared to freely available online information from the NHS **Moodzone** (See back page for more).

We would like to recruit over 2000 people from the whole of Nottinghamshire who have symptoms of anxiety and depression. Half of those who agree to take part will be given access to Big White Wall for 6 months, the other half will be provided with web links to the NHS Moodzone pages.

All participants will be asked to provide information about how they are feeling and other simple measures at particular time points over the course of 6 months.

Recruitment to REBOOT Notts is expected to run from late summer 2016 until summer 2018.

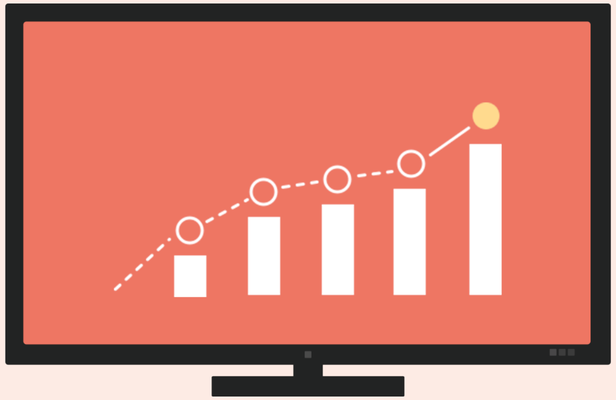

Why you might like to take part

If you are experiencing symptoms of anxiety and/or depression, such as feeling low, being uninterested in doing your usual activities, worrying a lot, having sleeping difficulties you may find that the information on the websites you access whilst participating in the study may be helpful.

It might also help you to understand how you are feeling and how your mood may change over time.

If you are not sure how you are feeling is associated with anxiety or depression, we will check this by using some established questionnaires before you join the study.

There are other things we will check to make sure you are suitable for the study. Many people find that being involved in a study can help them to feel positive about themselves by contributing to research that may help other people experiencing the same condition.

This study is being run by CLAHRC-EM (Collaboration for Leadership and Applied Health Research and Care – East Midlands) which is funded by the NHS to put research into practice as soon as possible if the findings are shown to be beneficial. This means that if you take part you may be helping us to help others in just a few years' time.

Rewards

Participants who complete their research measures at every stage will be entered into a prize draw to win £100 of high street vouchers, from which there will be 30 winners.

Whilst we do not pay people to participate in the research we hope that their involvement will enable them to access helpful information, record changes in their mood to help them understand how they are feeling as well feeling positive about taking part in research that will help local healthcare services to make informed decisions.

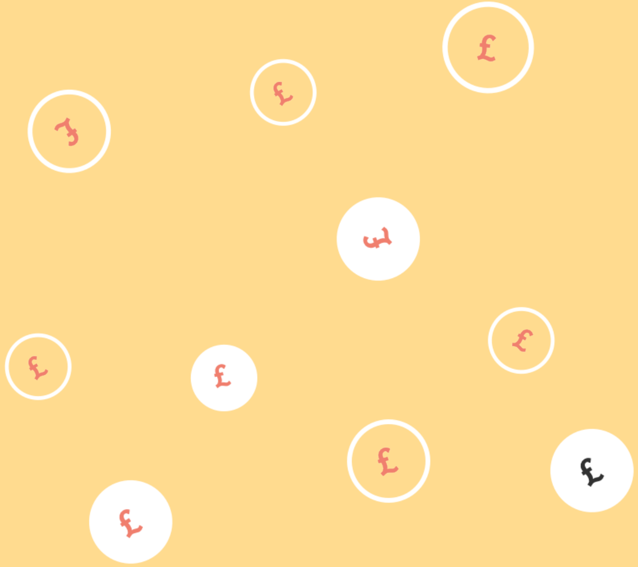

Frequently Asked Questions

Why might I be eligible?
You may be eligible to take part if you are:

- Experiencing low mood or anxiety
- Are over 16 years old
- Live in Nottinghamshire
- Have access to the internet

How long will my participation last?
You will be asked to remain in the study for 6 months and complete questionnaires about your mood at the start (baseline) 3, 6, 12 & 26 weeks.

How often do I need to access the Big White Wall or Moodzone?
It is entirely up to you how much or how little you access these other websites during the trial. You will have access to BWW for a period of 6 months from the start of your involvement in the study.

What will happen to my data?
Your data will be stored anonymously on a safe and secure server (approved by the NHS). It will be kept for research purposes for 7 years.

What if I no longer wish to take part in the study?
You may withdraw from the study at any time and without providing a reason and this will not affect your healthcare.

What if I have a problem with accessing the study site, BWW or Moodzone?
If you experience any technical difficulties, please contact the study team who will be able to advise you of what to do next.

MOODZONE

Moodzone deals with the kind of feelings and common life problems that affect lots of us from time to time.

Offers practical, useful information, interactive tools, and videos to support you on your way to feeling better.

Big White Wall™

BWW is designed to help patients get support, take control and feel better. It provides safe, anonymous peer and professional online support 24/7 (with trained wall-guides online at all times), plus a range of wellbeing tools to help people self-manage.

REBOOT Notts
Help us research online mental health support

This is a research study and participation is entirely voluntary.

This research is a project being run by:
Collaboration for Leadership In
Applied Health Research
and
Care (East Midlands) (CLAHRC-EM)

Scan the QR code, visit
www.rebootnotts.com
or email the research team at
reboot@nottingham.ac.uk

HRA REC 16/EM/0204 Leaflet Version 2 May 2017

REBOOT Notts

Feeling stressed or low?

Help us to research online support for wellbeing

www.rebootnotts.com

Or search for "Reboot Notts"

A partnership of:
The University of Nottingham, United Kingdom · China · Malaysia
University of Leicester
Nottinghamshire Healthcare NHS Foundation Trust
NHS National Institute for Health Research
Collaboration for Leadership in Applied Health Research and Care East Midlands
