# Supplementary material for: A Direct-to-Public Peer Support Program (Big White Wall) Versus Web-Based Information to Aid the Self-management of Depression and Anxiety: Results and Challenges of an Automated Randomized Controlled Trial
Source: J Med Internet Res. 2021 Apr 23;23(4):e23487. doi: 10.2196/23487 (PMC8105759; doi:10.2196/23487)
Supplement: Multimedia Appendix 2 [file jmir_v23i4e23487_app2.pdf]

Supplementary materials

Figure S1 Trifold leaflet (uploaded separately as a PDF)

Table S1. Unit costs, productivity resources, and disease prevalences.

Service	Unit cost (£)		Source
**Inpatient (per admittance)**			
Non elective inpatient	3117·75		NHS Reference costs 2017/18[1]
**Outpatient (per visit)**			
Accident and emergency	160·32		
Radiology	145·63		
Physiotherapist	54·91		
Occupational therapist	73·25		
Psychiatrist	353·05		
**Primary and community (per contact minute)**			PSSRU unit costs of health and social care 2018[2]
General practitioner	3·4		
GP home visit (excluding travel)	3·4		
Practical nurse	0·6		
Psychologist	1·47		
Psychiatric nurse	1·23		
Occupational therapist	0·72		
Social worker	0·73		
Out-of-hours care (per contact)	76·02		National Audit Office[3]
Walk-in centre (per contact)	76·02		
**Median team production multiplier**	1·28		Nicholson et al. (2006)[4]
**Median weekly pay - gross (£)**	**Male**	**Female**	Annual survey of hours and earnings, Office for National Statistics (2018)[5]
Full-time	621·18	519·28	
Part-time	180·85	193·70	
**Disease specific prevalence (%) 16-64**	5·8	9·48	Adult Psychiatric and Morbidity Survey, NHS Digital (2014)[6]
**Labour market activity (thousands) 16-64**	16326	14755	Labour market statistics time series - April 2019, Office for National Statistics (2019)[7]
**Working age population (thousands) 16-64**	20773	20873	Population estimates - mid-2018, Office for National Statistics (2019)[8]

IAPT cost used as primary outpatient referral pathway for anxiety and depression, incurs an initial fixed assessment cost of £121.08. Excludes qualification costs. Psychiatric nurses were costed as a band 6 community nurse. The cost-per-case, national average cost, for opted-out services was £68.30 2013 -> £76.02 in 2018 adjusted for inflation of 11.31% from ONS consumer price index. No individual costing information for walk-in centres was able to be located, this absence of literature was remarked by NICE[9], the sole evidence a comparative study which estimated a fixed cost of £76.54. Out-of- hours care costs were assumed a suitable proxy given the similarity between estimates and that walk-in centres were considered as a form of out-of-hours care[10]. Median salaries were adjusted for inflation (2%) using the change in consumer price index from June 2018 to June 2019[11].

References

1. NHS Improvement. NHS Reference Costs. 2018. https://improvement.nhs.uk/resources/reference-costs/
2. Curtis, L. & Burns, A. Unit Costs of Health and Social Care 2018, Personal Social Services Research Unit, University of Kent, Canterbury. 2018. https://doi.org/10.22024/UniKent/01.02.70995
3. National Audit Office. Out-of-hours GP services in England. 2014. https://www.nao.org.uk/wp-content/uploads/2014/09/Out-of-hours-GP-services-in-England1.pdf
4. Nicholson S, Pauly MV, Polsky D, Sharda C, Szrek H, Berger ML. Measuring the effects of work loss on productivity with team production. Health Econ. 2006. Feb;15(2):111-23. PubMed PMID: 16200550.

5. Office for National Statistics . Dataset: Earnings and hours worked, all employees: ASHE Table 1. 2018. https://www.ons.gov.uk/employmentandlabourmarket/peopleinwork/earningsandworkinghours/datasets/allemployeesashetable1
6. Health and Social Care Information Centre. Adult psychiatric and morbidity survey. 2014. https://digital.nhs.uk/data-and-information/publications/statistical/adult-psychiatric-morbidity-survey/adult-psychiatric-morbidity-survey-survey-of-mental-health-and-wellbeing-england-2014
7. Office for National Statistics. Dataset: Labour market statistics time series. 2019. https://www.ons.gov.uk/employmentandlabourmarket/peopleinwork/employmentandemployeetypes/datasets/labourmarketstatistics.
8. Office for National Statistics. Dataset: Estimates of the population for the UK, England and Wales, Scotland and Northern Ireland. 2019. https://www.ons.gov.uk/peoplepopulationandcommunity/populationandmigration/populationestimates/datasets/populationestimatesforukenglandandwalesscotlandandnorthernireland
9. National Institute for Health and Care Excellence (2018). Chapter 18 Minor injury unit, urgent care centre or walk-in centre. https://www.nice.org.uk/guidance/ng94/evidence/18.minor-injury-unit-urgent-care-centre-or-walkin-centre-pdf-172397464605
10. Salisbury et al. The Impact of co-located NHS walk-in centres on emergency departments. Emerg Med Journal 2007;24:265-269.
11. Office for National Statistics . Dataset: Consumer price inflation tables. 2019. https://www.ons.gov.uk/economy/inflationandpriceindices/datasets/consumerpriceinflation

Table S2. Summary of Engagement by researchers to recruit groups and services to help disseminate the study.

Adopter Type	Number contacted
Library	57
CityCare Teams	21
Allied Health Care Teams	39
Primary Schools (Staff and Parents)	248
Secondary Schools (staff and Parents)	65
Gyms	119
University Departments	119
Nurseries	151
Community Centres	183
Employers	219
Supermarkets	226
Foodbanks	31
Pharmacies	267
GP and Primary care Practices	175
Secondary Care Practices	42
Opinion Formers (e.g. CCGs)	12
Religious Organisations	18
Rural Organisations (e.g Young Farmers)	3
Third sector	79
Voluntary Organisations	76
General Marketing Campaign	
Door Drops and Leafleting	100k over the course of the study (2 x 10k door drops)
Facebook Advertising (Nativve)	3 x 1 month
Tram Advertising	1 x 8 weeks 1 x 4 weeks
Bus Advertising	2 x 4 weeks
Radio Interviews	2 x BBC Local Radio

Figures S2. Spread of randomised participants based on postcodes given.

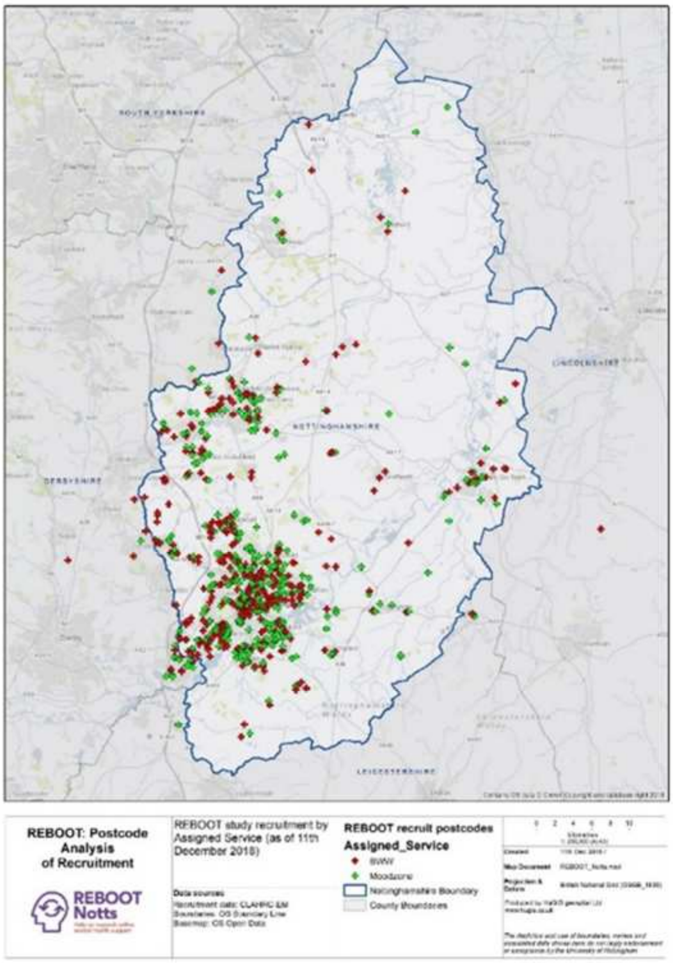

Figure S3 Spread of randomised participants recruited through Tram advertising (tram route in red).

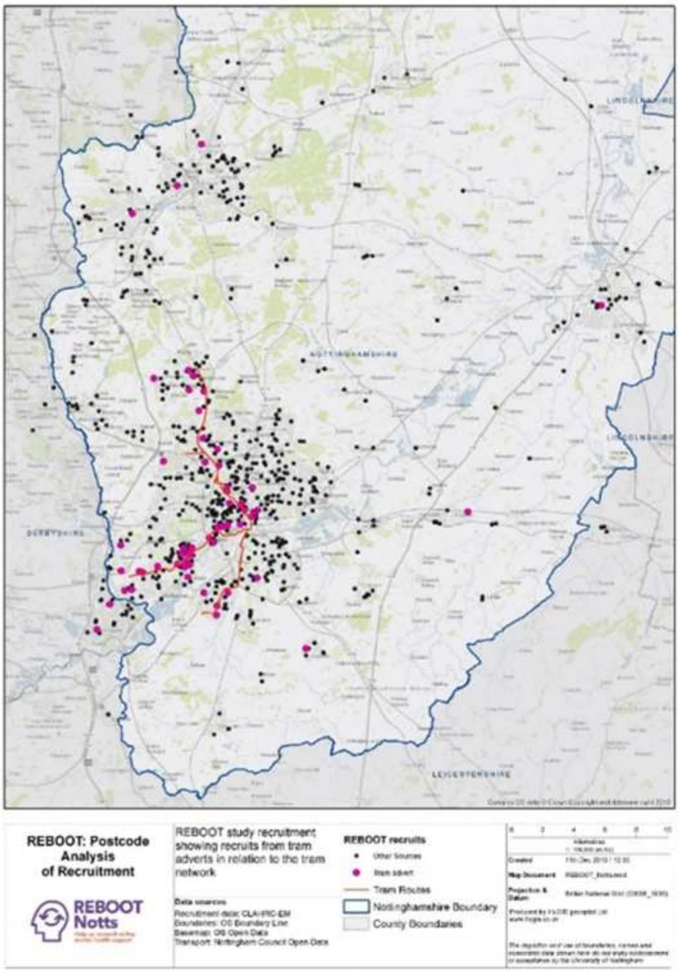

Table S3. Baseline self-reported intensity of service use at point of access within last three months.

Service	Arm 1 (*n*=397)			Arm 2 (*n*=393)		
**Inpatient**	*n/N*	Admissions	Bed days	*n/N*	Admissions	Bed days
General medical ward	7/7	1 (0)	5.5 (6.35)	8/8	1.5 (1.07)	5.86 (7.1)
Acute psychiatric ward	0/2	-	-	0/0	-	-
**Outpatient**		Visits			Visits	
A&E	20/20	1.35 (0.81)	-	18/18	1.7 (1.13)	-
Radiology	17/17	1.29 (0.69)	-	19/19	1.37 (0.6)	-
Physiotherapist	17/17	3.47 (3)	-	13/13	2.92 (1.7)	-
Occupational therapist	6/6	3.17 (4.4)	-	6/6	3.5 (3.51)	-
Psychiatrist	15/15	3.87 (4.19)	-	13/13	3.7 (4.77)	-
**Primary and community**		Contacts	Minutes		Contacts	Minutes
General practitioner	111/113	2.6 (1.64)	11.46 (4.9)	115/115	2.88 (2.09)	13.28 (10.68)
GP home visit	2/2	3.5 (3.55)	10 (0)	2/2	2.5 (0.71)	8.5 (9.19)
Practical nurse	31/31	1.55 (1.06)	11.1 (6.16)	38/38	1.95 (2.08)	12.92 (10.55)
Psychologist	5/6	5.8 (4.49)	44.33 (22.06)	3/3	8.67 (5.69)	46.67 (23.09)
Psychiatric Nurse	4/4	1.75 (0.98)	30 (0)	5/5	5.6 (5.86)	52.5 (15)
Occupational Therapist	2/2	4.5 (2.12)	82.5 (53.03)	6/6	1.33 (0.52)	40.83 (33.97)
Out-of-hours care	4/4	1.75 (1.5)	20.75 (12.74)	5/5	1.6 (0.55)	12.25 (6.85)
Walk-in centre	5/5	1.2 (0.48)	74 (78.21)	10/10	1.2 (0.42)	87.67 (96.6)
Social worker	1/1	3 (-)	60 (-)	2/2	2 (0)	120 (84.85)
