# Supplementary material for: A Direct-to-Public Peer Support Program (Big White Wall) Versus Web-Based Information to Aid the Self-management of Depression and Anxiety: Results and Challenges of an Automated Randomized Controlled Trial
Source: J Med Internet Res. 2021 Apr 23;23(4):e23487. doi: 10.2196/23487 (PMC8105759; doi:10.2196/23487)
Supplement: Multimedia Appendix 3 [file jmir_v23i4e23487_app3.pdf]

CONSORT-EHEALTH (V 1.6.1) - Submission/Publication Form

The CONSORT-EHEALTH checklist is intended for authors of randomized trials evaluating web-based and Internet-based applications/interventions, including mobile interventions, electronic games (incl multiplayer games), social media, certain telehealth applications, and other interactive and/or networked electronic applications. Some of the items (e.g. all subitems under item 5 - description of the intervention) may also be applicable for other study designs.

The goal of the CONSORT EHEALTH checklist and guideline is to be
a) a guide for reporting for authors of RCTs,
b) to form a basis for appraisal of an ehealth trial (in terms of validity)

CONSORT-EHEALTH items/subitems are MANDATORY reporting items for studies published in the Journal of Medical Internet Research and other journals / scientific societies endorsing the checklist.

Items numbered 1., 2., 3., 4a., 4b etc are original CONSORT or CONSORT-NPT (non-pharmacologic treatment) items.
Items with Roman numerals (i., ii, iii, iv etc.) are CONSORT-EHEALTH extensions/clarifications.

As the CONSORT-EHEALTH checklist is still considered in a formative stage, we would ask that you also RATE ON A SCALE OF 1-5 how important/useful you feel each item is FOR THE PURPOSE OF THE CHECKLIST and reporting guideline (optional).

Mandatory reporting items are marked with a red *.
In the textboxes, either copy & paste the relevant sections from your manuscript into this form - please include any quotes from your manuscript in QUOTATION MARKS,
or answer directly by providing additional information not in the manuscript, or elaborating on why the item was not relevant for this study.

YOUR ANSWERS WILL BE PUBLISHED AS A SUPPLEMENTARY FILE TO YOUR PUBLICATION IN JMIR AND ARE CONSIDERED PART OF YOUR PUBLICATION (IF ACCEPTED).
Please fill in these questions diligently. Information will not be copyedited, so please use proper spelling and grammar, use correct capitalization, and avoid abbreviations.

DO NOT FORGET TO SAVE AS PDF _AND_ CLICK THE SUBMIT BUTTON SO YOUR ANSWERS ARE IN OUR DATABASE !!!

Citation Suggestion (if you append the pdf as Appendix we suggest to cite this paper in the caption):
Eysenbach G, CONSORT-EHEALTH Group
CONSORT-EHEALTH: Improving and Standardizing Evaluation Reports of Web-based and Mobile Health Interventions
J Med Internet Res 2011;13(4):e126
URL: http://www.jmir.org/2011/4/e126/

doi: 10.2196/jmir.1923
PMID: 22209829

* Required

Your name *

First Last

Richard Morriss

Primary Affiliation (short), City, Country *

University of Toronto, Toronto, Canada

University of Nottingham, Nottingham, UK

Your e-mail address *

abc@gmail.com

richard.morriss@nottingham.ac.uk

Title of your manuscript *

Provide the (draft) title of your manuscript.

Outcomes of a public health campaign and automated randomised controlled trial of a direct to public peer support programme (Big White Wall) versus web-based information to aid self-management of depression and anxiety (The REBOOT study).

Name of your App/Software/Intervention *

If there is a short and a long/alternate name, write the short name first and add the long name in brackets.

Big White Wall versus Moodzone

Evaluated Version (if any)

e.g. "V1", "Release 2017-03-01", "Version 2.0.27913"

Your answer

Language(s) *

What language is the intervention/app in? If multiple languages are available, separate by comma (e.g. "English, French")

English

URL of your Intervention Website or App

e.g. a direct link to the mobile app on app in appstore (itunes, Google Play), or URL of the website. If the intervention is a DVD or hardware, you can also link to an Amazon page.

https://togetherall.com/en-gb/about-us/ and https://www.nhs.uk/conditions/stress-anxiety-c

URL of an image/screenshot (optional)

Your answer

Accessibility *

Can an enduser access the intervention presently?

- ○ access is free and open
- ○ access only for special usergroups, not open
- ◉ access is open to everyone, but requires payment/subscription/in-app purchases
- ○ app/intervention no longer accessible
- ○ Other:

Primary Medical Indication/Disease/Condition *

e.g. "Stress", "Diabetes", or define the target group in brackets after the condition, e.g. "Autism (Parents of children with)", "Alzheimers (Informal Caregivers of)"

Probable depression and/or anxiety

Primary Outcomes measured in trial *

comma-separated list of primary outcomes reported in the trial

14-item Warwick-Edinburgh Mental Well-Being

Secondary/other outcomes

Are there any other outcomes the intervention is expected to affect?

Patient Health Questionnaire (PHQ-9); General Anxiety Disorder scle (GAD-7); Work and Social Adjustment Scale (WSAS)

Recommended "Dose" *

What do the instructions for users say on how often the app should be used?

- ○ Approximately Daily
- ○ Approximately Weekly
- ○ Approximately Monthly
- ○ Approximately Yearly
- ◉ "as needed"
- ○ Other:

Approx. Percentage of Users (starters) still using the app as recommended after 3 months *

- (x) unknown / not evaluated
- ( ) 0-10%
- ( ) 11-20%
- ( ) 21-30%
- ( ) 31-40%
- ( ) 41-50%
- ( ) 51-60%
- ( ) 61-70%
- ( ) 71%-80%
- ( ) 81-90%
- ( ) 91-100%
- ( ) Other:

Overall, was the app/intervention effective? *

- ( ) yes: all primary outcomes were significantly better in intervention group vs control
- ( ) partly: SOME primary outcomes were significantly better in intervention group vs control
- ( ) no statistically significant difference between control and intervention
- ( ) potentially harmful: control was significantly better than intervention in one or more outcomes
- (x) inconclusive: more research is needed
- ( ) Other:

Article Preparation Status/Stage *

At which stage in your article preparation are you currently (at the time you fill in this form)

- ○ not submitted yet - in early draft status
- ○ not submitted yet - in late draft status, just before submission
- ◉ submitted to a journal but not reviewed yet
- ○ submitted to a journal and after receiving initial reviewer comments
- ○ submitted to a journal and accepted, but not published yet
- ○ published
- ○ Other:

Journal *

If you already know where you will submit this paper (or if it is already submitted), please provide the journal name (if it is not JMIR, provide the journal name under "other")

- ○ not submitted yet / unclear where I will submit this
- ◉ Journal of Medical Internet Research (JMIR)
- ○ JMIR mHealth and UHealth
- ○ JMIR Serious Games
- ○ JMIR Mental Health
- ○ JMIR Public Health
- ○ JMIR Formative Research
- ○ Other JMIR sister journal
- ○ Other:

Is this a full powered effectiveness trial or a pilot/feasibility trial? *

- ◯ Pilot/feasibility
- ◉ Fully powered

Manuscript tracking number *

If this is a JMIR submission, please provide the manuscript tracking number under "other" (The ms tracking number can be found in the submission acknowledgement email, or when you login as author in JMIR. If the paper is already published in JMIR, then the ms tracking number is the four-digit number at the end of the DOI, to be found at the bottom of each published article in JMIR)

- ◯ no ms number (yet) / not (yet) submitted to / published in JMIR
- ◉ Other: 23487

TITLE AND ABSTRACT

1a) TITLE: Identification as a randomized trial in the title

1a) Does your paper address CONSORT item 1a? *

I.e does the title contain the phrase "Randomized Controlled Trial"? (if not, explain the reason under "other")

- ◉ yes
- ◯ Other:

1a-i) Identify the mode of delivery in the title

Identify the mode of delivery. Preferably use “web-based” and/or “mobile” and/or “electronic game” in the title. Avoid ambiguous terms like “online”, “virtual”, “interactive”. Use “Internet-based” only if Intervention includes non-web-based Internet components (e.g. email), use “computer-based” or “electronic” only if offline products are used. Use “virtual” only in the context of “virtual reality” (3-D worlds). Use “online” only in the context of “online support groups”. Complement or substitute product names with broader terms for the class of products (such as “mobile” or “smart phone” instead of “iphone”), especially if the application runs on different platforms.

	1	2	3	4	5	
subitem not at all important	○	○	○	○	◉	essential

Clear selection

Does your paper address subitem 1a-i? *

Copy and paste relevant sections from manuscript title (include quotes in quotation marks "like this" to indicate direct quotes from your manuscript), or elaborate on this item by providing additional information not in the ms, or briefly explain why the item is not applicable/relevant for your study

"automated randomised controlled trial of a direct to public peer support programme (Big White Wall) versus web-based information to aid self-management of depression and anxiety"

1a-ii) Non-web-based components or important co-interventions in title

Mention non-web-based components or important co-interventions in title, if any (e.g., “with telephone support”).

	1	2	3	4	5	
subitem not at all important	○	○	○	○	◉	essential

Clear selection

Does your paper address subitem 1a-ii?

Copy and paste relevant sections from manuscript title (include quotes in quotation marks "like this" to indicate direct quotes from your manuscript), or elaborate on this item by providing additional information not in the ms, or briefly explain why the item is not applicable/relevant for your study

"automated randomised controlled trial of a direct to public peer support programme (Big White Wall) versus web-based information to aid self-management of depression and anxiety." Automated only.

1a-iii) Primary condition or target group in the title

Mention primary condition or target group in the title, if any (e.g., "for children with Type I Diabetes") Example: A Web-based and Mobile Intervention with Telephone Support for Children with Type I Diabetes: Randomized Controlled Trial

	1	2	3	4	5	
subitem not at all important	○	○	○	○	◉	essential

Clear selection

Does your paper address subitem 1a-iii? *

Copy and paste relevant sections from manuscript title (include quotes in quotation marks "like this" to indicate direct quotes from your manuscript), or elaborate on this item by providing additional information not in the ms, or briefly explain why the item is not applicable/relevant for your study

automated randomised controlled trial of a direct to public peer support programme (Big White Wall) versus web-based information to aid self-management of depression and anxiety

1b) ABSTRACT: Structured summary of trial design, methods, results, and conclusions

NPT extension: Description of experimental treatment, comparator, care providers, centers, and blinding status.

1b-i) Key features/functionalities/components of the intervention and comparator in the METHODS section of the ABSTRACT

Mention key features/functionalities/components of the intervention and comparator in the abstract. If possible, also mention theories and principles used for designing the site. Keep in mind the needs of systematic reviewers and indexers by including important synonyms. (Note: Only report in the abstract what the main paper is reporting. If this information is missing from the main body of text, consider adding it)

	1	2	3	4	5	
subitem not at all important	○	○	○	○	◉	essential

Clear selection

Does your paper address subitem 1b-i? *

Copy and paste relevant sections from the manuscript abstract (include quotes in quotation marks "like this" to indicate direct quotes from your manuscript), or elaborate on this item by providing additional information not in the ms, or briefly explain why the item is not applicable/relevant for your study

"A pragmatic, parallel group, single blind RCT was then conducted using a fully automated trial website in which eligible participants were randomised to receive either 6 months access to BWW or signposted to MZ". Theory behind design in published and reference protocol paper.

1b-ii) Level of human involvement in the METHODS section of the ABSTRACT

Clarify the level of human involvement in the abstract, e.g., use phrases like "fully automated" vs. "therapist/nurse/care provider/physician-assisted" (mention number and expertise of providers involved, if any). (Note: Only report in the abstract what the main paper is reporting. If this information is missing from the main body of text, consider adding it)

	1	2	3	4	5	
subitem not at all important	○	○	○	○	◉	essential

Clear selection

Does your paper address subitem 1b-ii?

Copy and paste relevant sections from the manuscript abstract (include quotes in quotation marks "like this" to indicate direct quotes from your manuscript), or elaborate on this item by providing additional information not in the ms, or briefly explain why the item is not applicable/relevant for your study

"A pragmatic, parallel group, single blind RCT was then conducted using a fully automated trial website in which eligible participants were randomised to receive either 6 months access to BWW or signposted to MZ." Fully automated trial.

1b-iii) Open vs. closed, web-based (self-assessment) vs. face-to-face assessments in the METHODS section of the ABSTRACT

Mention how participants were recruited (online vs. offline), e.g., from an open access website or from a clinic or a closed online user group (closed usergroup trial), and clarify if this was a purely web-based trial, or there were face-to-face components (as part of the intervention or for assessment). Clearly say if outcomes were self-assessed through questionnaires (as common in web-based trials). Note: In traditional offline trials, an open trial (open-label trial) is a type of clinical trial in which both the researchers and participants know which treatment is being administered. To avoid confusion, use “blinded” or “unblinded” to indicated the level of blinding instead of “open”, as “open” in web-based trials usually refers to “open access” (i.e. participants can self-enrol). (Note: Only report in the abstract what the main paper is reporting. If this information is missing from the main body of text, consider adding it)

	1	2	3	4	5	
subitem not at all important	○	○	○	○	◉	essential

Clear selection

Does your paper address subitem 1b-iii?

Copy and paste relevant sections from the manuscript abstract (include quotes in quotation marks "like this" to indicate direct quotes from your manuscript), or elaborate on this item by providing additional information not in the ms, or briefly explain why the item is not applicable/relevant for your study

"An 18 month campaign was performed across Nottinghamshire, United Kingdom (target population 914,000) to advertise the trial direct to the public through general marketing, online and social media sources, health services, other public services and third sector groups."
"The primary outcome was change in self-rated well-being at 6 weeks, measured by the Warwick-Edinburgh Mental Well-Being Scale."
"A pragmatic, parallel group, single blind RCT was then conducted using a fully automated trial website in which eligible participants were randomised to receive either 6 months access to BWW or signposted to MZ."
Statistician who carried out analysis of this otherwise fully automated trial was blinded (detail in main manuscript not abstract)

1b-iv) RESULTS section in abstract must contain use data

Report number of participants enrolled/assessed in each group, the use/uptake of the intervention (e.g., attrition/adherence metrics, use over time, number of logins etc.), in addition to primary/secondary outcomes. (Note: Only report in the abstract what the main paper is reporting. If this information is missing from the main body of text, consider adding it)

	1	2	3	4	5	
subitem not at all important	○	○	○	○	◉	essential

Clear selection

Does your paper address subitem 1b-iv?

Copy and paste relevant sections from the manuscript abstract (include quotes in quotation marks "like this" to indicate direct quotes from your manuscript), or elaborate on this item by providing additional information not in the ms, or briefly explain why the item is not applicable/relevant for your study

"Of 6483 visitors to the study website, only 1,510 were eligible. 790 (52.3% of eligible) participated, 397 randomised to BWW and 393 to MZ".

1b-v) CONCLUSIONS/DISCUSSION in abstract for negative trials

Conclusions/Discussions in abstract for negative trials: Discuss the primary outcome - if the trial is negative (primary outcome not changed), and the intervention was not used, discuss whether negative results are attributable to lack of uptake and discuss reasons. (Note: Only report in the abstract what the main paper is reporting. If this information is missing from the main body of text, consider adding it)

	1	2	3	4	5	
subitem not at all important	○	○	○	○	◉	essential

Clear selection

Does your paper address subitem 1b-v?

Copy and paste relevant sections from the manuscript abstract (include quotes in quotation marks "like this" to indicate direct quotes from your manuscript), or elaborate on this item by providing additional information not in the ms, or briefly explain why the item is not applicable/relevant for your study

"A fully automated RCT recruiting directly from the public failed to recruit and retain sufficient participants to test the clinical effectiveness of this digital intervention."

INTRODUCTION

2a) In INTRODUCTION: Scientific background and explanation of rationale

2a-i) Problem and the type of system/solution

Describe the problem and the type of system/solution that is object of the study: intended as stand-alone intervention vs. incorporated in broader health care program? Intended for a particular patient population? Goals of the intervention, e.g., being more cost-effective to other interventions, replace or complement other solutions? (Note: Details about the intervention are provided in “Methods” under 5)

	1	2	3	4	5	
subitem not at all important	○	○	○	○	◉	essential

Clear selection

Does your paper address subitem 2a-i? *

Copy and paste relevant sections from the manuscript (include quotes in quotation marks "like this" to indicate direct quotes from your manuscript), or elaborate on this item by providing additional information not in the ms, or briefly explain why the item is not applicable/relevant for your study

"In principle, the internet backed by a public health campaign might be a useful platform for reaching people with depression or anxiety who do not or are unable to access face-to-face health care. Yet, use of the internet as a potential therapeutic platform raises a series of important concerns about data safety and privacy, effectiveness, user experience/adherence, exclusion of people without access to the internet, and data integration with care [3,4]. It is important to establish who might be reached by such an approach, whether or not they are already accessing mental health or general health services, and which recruitment approaches and interventions are most effective [5]".
"To date, BWW and other online peer support for depression or anxiety have only been evaluated in RCTs conducted through primary or specialist mental health services rather than direct to the public [10-13]. There is uncertainty around how to conduct fully automated randomised controlled trials (RCTs) of digital mental health interventions directly targeted at the public [14]. In principle, fully automated RCTs without human contact are less prone to bias and can better elucidate actual treatment effects attributable to the digital intervention than those conducted with human conduct, where the human contact may contribute to part of the treatment effect [15]. They could also be relatively inexpensive to run on a large scale [16]. However, it is unclear if and under what circumstances they would be feasible or acceptable to participants [17]. Automated trials have sometimes failed to engage some populations [18, 19] while others offering otherwise difficult to obtain structured psychological treatments have been more successful [20-22]".

2a-ii) Scientific background, rationale: What is known about the (type of) system

Scientific background, rationale: What is known about the (type of) system that is the object of the study (be sure to discuss the use of similar systems for other conditions/diagnoses, if appropiate), motivation for the study, i.e. what are the reasons for and what is the context for this specific study, from which stakeholder viewpoint is the study performed, potential impact of findings [2]. Briefly justify the choice of the comparator.

	1	2	3	4	5	
subitem not at all important	○	○	○	○	◉	essential

Clear selection

Does your paper address subitem 2a-ii? *

Copy and paste relevant sections from the manuscript (include quotes in quotation marks "like this" to indicate direct quotes from your manuscript), or elaborate on this item by providing additional information not in the ms, or briefly explain why the item is not applicable/relevant for your study

"To date, BWW and other online peer support for depression or anxiety have only been evaluated in RCTs conducted through primary or specialist mental health services rather than direct to the public [10-13]. There is uncertainty around how to conduct fully automated randomised controlled trials (RCTs) of digital mental health interventions directly targeted at the public [14]. In principle, fully automated RCTs without human contact are less prone to bias and can better elucidate actual treatment effects attributable to the digital intervention than those conducted with human conduct, where the human contact may contribute to part of the treatment effect [15]. They could also be relatively inexpensive to run on a large scale [16]. However, it is unclear if and under what circumstances they would be feasible or acceptable to participants [17]. Automated trials have sometimes failed to engage some populations [18, 19] while others offering otherwise difficult to obtain structured psychological treatments have been more successful [20-22]."
More detail on the development of BWW in published and referenced trial protocol paper.

2b) In INTRODUCTION: Specific objectives or hypotheses

Does your paper address CONSORT subitem 2b? *

Copy and paste relevant sections from the manuscript (include quotes in quotation marks "like this" to indicate direct quotes from your manuscript), or elaborate on this item by providing additional information not in the ms, or briefly explain why the item is not applicable/relevant for your study

"The current study had two main aims:
1. To investigate the reach, feasibility and acceptability of a public health recruitment campaign using general media, digital media, health, third sector and social services for a trial in people with probable mild to moderate depression or anxiety.
2. To test the feasibility, acceptability, baseline costs to society and outcomes of conducting a fully automated RCT of two established digital interventions providing moderated online peer support and information (BWW) versus online information only (MZ)."

METHODS

3a) Description of trial design (such as parallel, factorial) including allocation ratio

Does your paper address CONSORT subitem 3a? *

Copy and paste relevant sections from the manuscript (include quotes in quotation marks "like this" to indicate direct quotes from your manuscript), or elaborate on this item by providing additional information not in the ms, or briefly explain why the item is not applicable/relevant for your study

"The second stage of the study was a single-blind, randomised controlled feasibility trial (RCT) using a fully automated bespoke study website. Full details are available in the published protocol [24]. Eligible participants self-referred and were recruited through the study website following the public health campaign. Consenting participants were allocated at random to receive either six months free access to BWW or signposted to the NHS Moodzone (MZ) website." Randomisation allocation of 1:1 demonstrated in CONSORT figure showing flow through the study.

3b) Important changes to methods after trial commencement (such as eligibility criteria), with reasons

Does your paper address CONSORT subitem 3b? *

Copy and paste relevant sections from the manuscript (include quotes in quotation marks "like this" to indicate direct quotes from your manuscript), or elaborate on this item by providing additional information not in the ms, or briefly explain why the item is not applicable/relevant for your study

"Feedback left by the first 50 participants suggested they disliked the intrusiveness and length of some of the measures and the assessments at baseline. One participant withdrew from the study for this reason. Therefore, compared to our protocol [24], we omitted the 12-item medical outcomes study short form health survey version 2.0 (SF-12v2) [34] at all time points and only carried out the economic resource proforma [31] at baseline. At baseline the number of questions asked fell from 92 to 80, and at each follow up time point from 50 to 38."

3b-i) Bug fixes, Downtimes, Content Changes

Bug fixes, Downtimes, Content Changes: ehealth systems are often dynamic systems. A description of changes to methods therefore also includes important changes made on the intervention or comparator during the trial (e.g., major bug fixes or changes in the functionality or content) (5-iii) and other “unexpected events” that may have influenced study design such as staff changes, system failures/downtimes, etc. [2].

	1	2	3	4	5	
subitem not at all important	○	○	○	○	●	essential

Clear selection

Does your paper address subitem 3b-i?

Copy and paste relevant sections from the manuscript (include quotes in quotation marks "like this" to indicate direct quotes from your manuscript), or elaborate on this item by providing additional information not in the ms, or briefly explain why the item is not applicable/relevant for your study

No bug fixes or content changes. "The study experienced technical issues, such as website downtime, problems with progression through the site and errors within the measures, which may have deterred completion of some measures and retention in the study."

4a) Eligibility criteria for participants

Does your paper address CONSORT subitem 4a? *

Copy and paste relevant sections from the manuscript (include quotes in quotation marks "like this" to indicate direct quotes from your manuscript), or elaborate on this item by providing additional information not in the ms, or briefly explain why the item is not applicable/relevant for your study

"Inclusion criteria were:
• Aged 16 years or over
• Resident in the County of Nottinghamshire
• Scores between 10-20 on the 9-Item Personal Health Questionnaire (PHQ-9) [25] or 10 or more on the 7-Item Generalised Anxiety Disorder questionnaire (GAD-7) [26], indicating probable caseness for depression and anxiety respectively but not a definite diagnosis of depression or anxiety disorder
• Access to internet through a computer, tablet or smartphone (Windows, iOS, Android) and email address.
• Able and willing to give informed consent (through electronic consent)

Exclusion Criteria:
• Scores >21 on the PHQ-9 (severe depression)
• Scores 2 or 3 on PHQ-9 item "thoughts that you would be better off dead or of hurting yourself in some way".
• Scores <10 on PHQ-9 and GAD-7
• BWW and Moodzone are only available in English. Therefore the website recommended non-participation for those who believed they were insufficiently proficient in the use of the English language ."

4a-i) Computer / Internet literacy

Computer / Internet literacy is often an implicit “de facto” eligibility criterion - this should be explicitly clarified.

	1	2	3	4	5	
subitem not at all important	○	○	○	○	◉	essential

Clear selection

Does your paper address subitem 4a-i?

Copy and paste relevant sections from the manuscript (include quotes in quotation marks "like this" to indicate direct quotes from your manuscript), or elaborate on this item by providing additional information not in the ms, or briefly explain why the item is not applicable/relevant for your study

There was no test of proficiency in English or information technology literacy.

4a-ii) Open vs. closed, web-based vs. face-to-face assessments:

Open vs. closed, web-based vs. face-to-face assessments: Mention how participants were recruited (online vs. offline), e.g., from an open access website or from a clinic, and clarify if this was a purely web-based trial, or there were face-to-face components (as part of the intervention or for assessment), i.e., to what degree got the study team to know the participant. In online-only trials, clarify if participants were quasi-anonymous and whether having multiple identities was possible or whether technical or logistical measures (e.g., cookies, email confirmation, phone calls) were used to detect/prevent these.

	1	2	3	4	5	
subitem not at all important	○	○	○	○	◉	essential

Clear selection

Does your paper address subitem 4a-ii? *

Copy and paste relevant sections from the manuscript (include quotes in quotation marks "like this" to indicate direct quotes from your manuscript), or elaborate on this item by providing additional information not in the ms, or briefly explain why the item is not applicable/relevant for your study

"We ran a public health campaign using these terms and offering the opportunity to take part in a study free of charge comparing an information-giving website (MZ) that is a standard designed with and used within the English NHS, with an online peer support site (BWW). We used a mix of traditional health research recruitment strategies, such as GP endorsement, outpatient clinics and support groups as well as less traditional advertising, such as on buses and trams and via letter box leafleting. Special effort was made to reach groups regarded as higher risk and harder to reach, e.g. the farming community."
"The second stage of the study was a single-blind, randomised controlled feasibility trial (RCT) using a fully automated bespoke study website. Full details are available in the published protocol [24]. Eligible participants self-referred and were recruited through the study website following the public health campaign. Consenting participants were allocated at random to receive either six months free access to BWW or signposted to the NHS Moodzone (MZ) website."
" Potential participants from the county of Nottinghamshire self-referred to the study and their eligibility was assessed by an automated digital programme on the study website. "
"Once consented, participants were asked to complete self-rated questionnaires to measure well-being, depression, anxiety, work and social adjustment, receipt of services (for economic analysis), social support, and personality dysfunction at baseline. These were completed online (though the study website) in approximately 20-30 minutes. All data was stored on the website and downloaded and anonymised by the clinical trial manager.
Participation in the study was for six months. Participants received electronic follow up invitations at 3, 6, 12 and 26 weeks after randomisation to be completed on the website. Each participant was reminded to log onto the study website and complete follow up measures by email 24 hours before each follow up and at the follow up time point. If follow up was not completed, they received another reminder 48 hours later. Participants were emailed motivational statements encouraging follow up as well as the offer of entry into a prize draw at the end of the study if they completed at least the primary outcome measure in all follow up assessments. There were no other attempts to follow up participants using any form of digital, telephone or face to face contact."

4a-iii) Information giving during recruitment

Information given during recruitment. Specify how participants were briefed for recruitment and in the informed consent procedures (e.g., publish the informed consent documentation as appendix, see also item X26), as this information may have an effect on user self-selection, user expectation and may also bias results.

	1	2	3	4	5	
subitem not at all important	○	○	○	○	◉	essential

Clear selection

Does your paper address subitem 4a-iii?

Copy and paste relevant sections from the manuscript (include quotes in quotation marks "like this" to indicate direct quotes from your manuscript), or elaborate on this item by providing additional information not in the ms, or briefly explain why the item is not applicable/relevant for your study

"Information for participants and the associated consent forms were provided electronically within the study website. Participants who wished to discuss the study could email and telephone the study team if they had any further questions before consenting to the study. An email confirming consent was sent to each participant once they had fully enrolled."

4b) Settings and locations where the data were collected

Does your paper address CONSORT subitem 4b? *

Copy and paste relevant sections from the manuscript (include quotes in quotation marks "like this" to indicate direct quotes from your manuscript), or elaborate on this item by providing additional information not in the ms, or briefly explain why the item is not applicable/relevant for your study

Your answer

4b-i) Report if outcomes were (self-)assessed through online questionnaires

Clearly report if outcomes were (self-)assessed through online questionnaires (as common in web-based trials) or otherwise.

	1	2	3	4	5	
subitem not at all important	○	○	○	○	◉	essential

Clear selection

Does your paper address subitem 4b-i? *

Copy and paste relevant sections from the manuscript (include quotes in quotation marks "like this" to indicate direct quotes from your manuscript), or elaborate on this item by providing additional information not in the ms, or briefly explain why the item is not applicable/relevant for your study

"Once consented, participants were asked to complete self-rated questionnaires to measure well-being, depression, anxiety, work and social adjustment, receipt of services (for economic analysis), social support, and personality dysfunction at baseline. These were completed online (though the study website) in approximately 20-30 minutes. All data was stored on the website and downloaded and anonymised by the clinical trial manager."
"Participation in the study was for six months. Participants received electronic follow up invitations at 3, 6, 12 and 26 weeks after randomisation to be completed on the website. Each participant was reminded to log onto the study website and complete follow up measures by email 24 hours before each follow up and at the follow up time point. If follow up was not completed, they received another reminder 48 hours later. Participants were emailed motivational statements encouraging follow up as well as the offer of entry into a prize draw at the end of the study if they completed at least the primary outcome measure in all follow up assessments. There were no other attempts to follow up participants using any form of digital, telephone or face to face contact. "

4b-ii) Report how institutional affiliations are displayed

Report how institutional affiliations are displayed to potential participants [on ehealth media], as affiliations with prestigious hospitals or universities may affect volunteer rates, use, and reactions with regards to an intervention.(Not a required item – describe only if this may bias results)

	1	2	3	4	5	
subitem not at all important	○	○	◉	○	○	essential

Clear selection

Does your paper address subitem 4b-ii?

Copy and paste relevant sections from the manuscript (include quotes in quotation marks "like this" to indicate direct quotes from your manuscript), or elaborate on this item by providing additional information not in the ms, or briefly explain why the item is not applicable/relevant for your study

All publicity indicated that the study was funded by NIHR CLAHRC East Midlands and conducted by the University of Nottingham. We were advised by our LIved Experience Advisory Panel on the wording of such publicity including what institutional affiliations to report and their prominence in the publicity material.

5) The interventions for each group with sufficient details to allow replication, including how and when they were actually administered

5-i) Mention names, credential, affiliations of the developers, sponsors, and owners

Mention names, credential, affiliations of the developers, sponsors, and owners [6] (if authors/evaluators are owners or developer of the software, this needs to be declared in a "Conflict of interest" section or mentioned elsewhere in the manuscript).

	1	2	3	4	5	
subitem not at all important	○	○	○	○	◉	essential

Clear selection

Does your paper address subitem 5-i?

Copy and paste relevant sections from the manuscript (include quotes in quotation marks "like this" to indicate direct quotes from your manuscript), or elaborate on this item by providing additional information not in the ms, or briefly explain why the item is not applicable/relevant for your study

"Randomisation: Arm 1: Big White Wall
Participants allocated to receive six months free access to the Big White Wall website [8] were invited to create a user profile using a pseudonym which was linked to the trial identification to which they had been assigned within the study website. They had to do this within 14 days of being randomised. Participants were able to access any part of the BWW site (apart from the option of personalised therapy or counselling sessions which have to be prescribed by a clinician i.e. not offered direct to the general public), and interact with other users within the boundaries of the site's House Rules. Anonymised records of logins, time on site, interactions and page categories were recorded by BWW on behalf of the study team.
Randomisation: Arm 2: Participants allocated to Moodzone
Participants were directed to the Moodzone area of the NHS Choices website [9]. Participants were able to access all the available material on mental health including depression and anxiety. We did not have records of time on site or use of the site. NHS MZ access was used as the control digital resource so all participants were offered some help for their problems with depression or anxiety but this control group did not have access to moderated, anonymised peer social support."
"Figure 1 shows that there were 6483 visitors to the study website (14 per day) over 18 months of recruitment from September 16th 2016 to May 30th 2018. "

5-ii) Describe the history/development process

Describe the history/development process of the application and previous formative evaluations (e.g., focus groups, usability testing), as these will have an impact on adoption/use rates and help with interpreting results.

	1	2	3	4	5	
subitem not at all important	○	○	○	◉	○	essential

Clear selection

Does your paper address subitem 5-ii?

Copy and paste relevant sections from the manuscript (include quotes in quotation marks "like this" to indicate direct quotes from your manuscript), or elaborate on this item by providing additional information not in the ms, or briefly explain why the item is not applicable/relevant for your study

"A Lived Experience Advisory Panel of patients and public representatives with personal experience of depression and anxiety that was formed for the study advised us on the best approaches to recruiting people with depression and anxiety in the community. They recommended using the terms “low in mood” for depression and “stressed” for anxiety. We ran a public health campaign using these terms and offering the opportunity to take part in a study free of charge comparing an information-giving website (MZ) that is a standard designed with and used within the English NHS, with an online peer support site (BWW). We used a mix of traditional health research recruitment strategies, such as GP endorsement, outpatient clinics and support groups as well as less traditional advertising, such as on buses and trams and via letter box leafleting. Special effort was made to reach groups regarded as higher risk and harder to reach, e.g. the farming community. Reach was defined as the absolute number, proportion, and representativeness of individuals who were willing to participate in a given initiative [23]."
"The research team worked closely with a research delivery and support service (the National Insitute of Health Research (NIHR) Clinical Research Network (CRN) East Midlands), a professional marketing business (The Dairy), the study Lived Experience Advisory Panel (LEAP) (18 people aged 25 to 65 years, 12 female) and the web developer (Ayup). The aim was to establish a brand for the study that was considered by the LEAP and study team to appeal to people who may be suffering from low mood and/or stress in line with marketing materials, but also instilled professionalism and confidence with respect to the research project."
More detail on the development of Big White Wall is given in the published and referenced trial protocol paper.

5-iii) Revisions and updating

Revisions and updating. Clearly mention the date and/or version number of the application/intervention (and comparator, if applicable) evaluated, or describe whether the intervention underwent major changes during the evaluation process, or whether the development and/or content was “frozen” during the trial. Describe dynamic components such as news feeds or changing content which may have an impact on the replicability of the intervention (for unexpected events see item 3b).

	1	2	3	4	5	
subitem not at all important	○	○	○	◉	○	essential

Clear selection

Does your paper address subitem 5-iii?

Copy and paste relevant sections from the manuscript (include quotes in quotation marks "like this" to indicate direct quotes from your manuscript), or elaborate on this item by providing additional information not in the ms, or briefly explain why the item is not applicable/relevant for your study

"Figure 1 shows that there were 6483 visitors to the study website (14 per day) over 18 months of recruitment from September 16th 2016 to May 30th 2018." There were no revisions or updates during the trial.

5-iv) Quality assurance methods

Provide information on quality assurance methods to ensure accuracy and quality of information provided [1], if applicable.

	1	2	3	4	5	
subitem not at all important	○	○	○	○	◉	essential

Clear selection

Does your paper address subitem 5-iv?

Copy and paste relevant sections from the manuscript (include quotes in quotation marks "like this" to indicate direct quotes from your manuscript), or elaborate on this item by providing additional information not in the ms, or briefly explain why the item is not applicable/relevant for your study

"These were completed online (though the study website) in approximately 20-30 minutes. All data was stored on the website and downloaded and anonymised by the clinical trial manager. Participation in the study was for six months. Participants received electronic follow up invitations at 3, 6, 12 and 26 weeks after randomisation to be completed on the website. Each participant was reminded to log onto the study website and complete follow up measures by email 24 hours before each follow up and at the follow up time point. If follow up was not completed, they received another reminder 48 hours later. Participants were emailed motivational statements encouraging follow up as well as the offer of entry into a prize draw at the end of the study if they completed at least the primary outcome measure in all follow up assessments. There were no other attempts to follow up participants using any form of digital, telephone or face to face contact."
"All the analysis was performed on an intention to treat basis by the trial statistician blinded to treatment allocation using STATA 16. "

5-v) Ensure replicability by publishing the source code, and/or providing screenshots/screen-capture video, and/or providing flowcharts of the algorithms used

Ensure replicability by publishing the source code, and/or providing screenshots/screen-capture video, and/or providing flowcharts of the algorithms used. Replicability (i.e., other researchers should in principle be able to replicate the study) is a hallmark of scientific reporting.

	1	2	3	4	5	
subitem not at all important	○	○	●	○	○	essential

Clear selection

Does your paper address subitem 5-v?

Copy and paste relevant sections from the manuscript (include quotes in quotation marks "like this" to indicate direct quotes from your manuscript), or elaborate on this item by providing additional information not in the ms, or briefly explain why the item is not applicable/relevant for your study

The interventions are interactive websites for which URLs are provided. We do not own the IP so cannot give algorithms and a snapshot or video would also not be informative.

5-vi) Digital preservation

Digital preservation: Provide the URL of the application, but as the intervention is likely to change or disappear over the course of the years; also make sure the intervention is archived (Internet Archive, webcitation.org, and/or publishing the source code or screenshots/videos alongside the article). As pages behind login screens cannot be archived, consider creating demo pages which are accessible without login.

	1	2	3	4	5	
subitem not at all important	○	○	●	○	○	essential

Clear selection

Does your paper address subitem 5-vi?

Copy and paste relevant sections from the manuscript (include quotes in quotation marks "like this" to indicate direct quotes from your manuscript), or elaborate on this item by providing additional information not in the ms, or briefly explain why the item is not applicable/relevant for your study

The interventions are interactive websites for which URLs are provided. We do not own the IP so cannot give algorithms and a snapshot or video would also not be informative. We give the dates of recruitment into the study (see previous answers).

5-vii) Access

Access: Describe how participants accessed the application, in what setting/context, if they had to pay (or were paid) or not, whether they had to be a member of specific group. If known, describe how participants obtained "access to the platform and Internet" [1]. To ensure access for editors/reviewers/readers, consider to provide a "backdoor" login account or demo mode for reviewers/readers to explore the application (also important for archiving purposes, see vi).

	1	2	3	4	5	
subitem not at all important	○	○	○	○	◉	essential

Clear selection

Does your paper address subitem 5-vii? *

Copy and paste relevant sections from the manuscript (include quotes in quotation marks "like this" to indicate direct quotes from your manuscript), or elaborate on this item by providing additional information not in the ms, or briefly explain why the item is not applicable/relevant for your study

"The second stage of the study was a single-blind, randomised controlled feasibility trial (RCT) using a fully automated bespoke study website. Full details are available in the published protocol [24]. Eligible participants self-referred and were recruited through the study website following the public health campaign. Consenting participants were allocated at random to receive either six months free access to BWW or signposted to the NHS Moodzone (MZ) website."

5-viii) Mode of delivery, features/functionalities/components of the intervention and comparator, and the theoretical framework

Describe mode of delivery, features/functionalities/components of the intervention and comparator, and the theoretical framework [6] used to design them (instructional strategy [1], behaviour change techniques, persuasive features, etc., see e.g., [7, 8] for terminology). This includes an in-depth description of the content (including where it is coming from and who developed it) [1]," whether [and how] it is tailored to individual circumstances and allows users to track their progress and receive feedback" [6]. This also includes a description of communication delivery channels and – if computer-mediated communication is a component – whether communication was synchronous or asynchronous [6]. It also includes information on presentation strategies [1], including page design principles, average amount of text on pages, presence of hyperlinks to other resources, etc. [1].

	1	2	3	4	5	
subitem not at all important	○	○	○	◉	○	essential

Clear selection

Does your paper address subitem 5-viii? *

Copy and paste relevant sections from the manuscript (include quotes in quotation marks "like this" to indicate direct quotes from your manuscript), or elaborate on this item by providing additional information not in the ms, or briefly explain why the item is not applicable/relevant for your study

"Randomisation: Arm 1: Big White Wall
Participants allocated to receive six months free access to the Big White Wall website [8] were invited to create a user profile using a pseudonym which was linked to the trial identification to which they had been assigned within the study website. They had to do this within 14 days of being randomised. Participants were able to access any part of the BWW site (apart from the option of personalised therapy or counselling sessions which have to be prescribed by a clinician i.e. not offered direct to the general public), and interact with other users within the boundaries of the site's House Rules. Anonymised records of logins, time on site, interactions and page categories were recorded by BWW on behalf of the study team.
Randomisation: Arm 2: Participants allocated to Moodzone
Participants were directed to the Moodzone area of the NHS Choices website [9]. Participants were able to access all the available material on mental health including depression and anxiety. We did not have records of time on site or use of the site. NHS MZ access was used as the control digital resource so all participants were offered some help for their problems with depression or anxiety but this control group did not have access to moderated, anonymised peer social support."
More detail on the development and theory behind these interventions is given in the published and referenced trial protocol paper.

5-ix) Describe use parameters

Describe use parameters (e.g., intended "doses" and optimal timing for use). Clarify what instructions or recommendations were given to the user, e.g., regarding timing, frequency, heaviness of use, if any, or was the intervention used ad libitum.

	1	2	3	4	5	
subitem not at all important	○	○	○	○	◉	essential

Clear selection

Does your paper address subitem 5-ix?

Copy and paste relevant sections from the manuscript (include quotes in quotation marks "like this" to indicate direct quotes from your manuscript), or elaborate on this item by providing additional information not in the ms, or briefly explain why the item is not applicable/relevant for your study

"Randomisation: Arm 1: Big White Wall
Participants allocated to receive six months free access to the Big White Wall website [8] were invited to create a user profile using a pseudonym which was linked to the trial identification to which they had been assigned within the study website. They had to do this within 14 days of being randomised. Participants were able to access any part of the BWW site (apart from the option of personalised therapy or counselling sessions which have to be prescribed by a clinician i.e. not offered direct to the general public), and interact with other users within the boundaries of the site's House Rules. Anonymised records of logins, time on site, interactions and page categories were recorded by BWW on behalf of the study team.
Randomisation: Arm 2: Participants allocated to Moodzone
Participants were directed to the Moodzone area of the NHS Choices website [9]. Participants were able to access all the available material on mental health including depression and anxiety. We did not have records of time on site or use of the site. NHS MZ access was used as the control digital resource so all participants were offered some help for their problems with depression or anxiety but this control group did not have access to moderated, anonymised peer social support."

5-x) Clarify the level of human involvement

Clarify the level of human involvement (care providers or health professionals, also technical assistance) in the e-intervention or as co-intervention (detail number and expertise of professionals involved, if any, as well as "type of assistance offered, the timing and frequency of the support, how it is initiated, and the medium by which the assistance is delivered". It may be necessary to distinguish between the level of human involvement required for the trial, and the level of human involvement required for a routine application outside of a RCT setting (discuss under item 21 – generalizability).

	1	2	3	4	5	
subitem not at all important	○	○	○	○	◉	essential

Clear selection

Does your paper address subitem 5-x?

Copy and paste relevant sections from the manuscript (include quotes in quotation marks "like this" to indicate direct quotes from your manuscript), or elaborate on this item by providing additional information not in the ms, or briefly explain why the item is not applicable/relevant for your study

"Potential participants from the county of Nottinghamshire self-referred to the study and their eligibility was assessed by an automated digital programme on the study website. "
"Information for participants and the associated consent forms were provided electronically within the study website. Participants who wished to discuss the study could email and telephone the study team if they had any further questions before consenting to the study. An email confirming consent was sent to each participant once they had fully enrolled."
"Once consented, participants were asked to complete self-rated questionnaires to measure well-being, depression, anxiety, work and social adjustment, receipt of services (for economic analysis), social support, and personality dysfunction at baseline. These were completed online (though the study website) in approximately 20-30 minutes. All data was stored on the website and downloaded and anonymised by the clinical trial manager.
Participation in the study was for six months. Participants received electronic follow up invitations at 3, 6, 12 and 26 weeks after randomisation to be completed on the website. Each participant was reminded to log onto the study website and complete follow up measures by email 24 hours before each follow up and at the follow up time point. If follow up was not completed, they received another reminder 48 hours later. Participants were emailed motivational statements encouraging follow up as well as the offer of entry into a prize draw at the end of the study if they completed at least the primary outcome measure in all follow up assessments. There were no other attempts to follow up participants using any form of digital, telephone or face to face contact."

5-xi) Report any prompts/reminders used

Report any prompts/reminders used: Clarify if there were prompts (letters, emails, phone calls, SMS) to use the application, what triggered them, frequency etc. It may be necessary to distinguish between the level of prompts/reminders required for the trial, and the level of prompts/reminders for a routine application outside of a RCT setting (discuss under item 21 – generalizability).

	1	2	3	4	5	
subitem not at all important	○	○	○	○	◉	essential

Clear selection

Does your paper address subitem 5-xi? *

Copy and paste relevant sections from the manuscript (include quotes in quotation marks "like this" to indicate direct quotes from your manuscript), or elaborate on this item by providing additional information not in the ms, or briefly explain why the item is not applicable/relevant for your study

"Participation in the study was for six months. Participants received electronic follow up invitations at 3, 6, 12 and 26 weeks after randomisation to be completed on the website. Each participant was reminded to log onto the study website and complete follow up measures by email 24 hours before each follow up and at the follow up time point. If follow up was not completed, they received another reminder 48 hours later. Participants were emailed motivational statements encouraging follow up as well as the offer of entry into a prize draw at the end of the study if they completed at least the primary outcome measure in all follow up assessments. There were no other attempts to follow up participants using any form of digital, telephone or face to face contact."

5-xii) Describe any co-interventions (incl. training/support)

Describe any co-interventions (incl. training/support): Clearly state any interventions that are provided in addition to the targeted eHealth intervention, as ehealth intervention may not be designed as stand-alone intervention. This includes training sessions and support [1]. It may be necessary to distinguish between the level of training required for the trial, and the level of training for a routine application outside of a RCT setting (discuss under item 21 – generalizability.

	1	2	3	4	5	
subitem not at all important	○	○	○	○	◉	essential

Clear selection

Does your paper address subitem 5-xii? *

Copy and paste relevant sections from the manuscript (include quotes in quotation marks "like this" to indicate direct quotes from your manuscript), or elaborate on this item by providing additional information not in the ms, or briefly explain why the item is not applicable/relevant for your study

"Randomisation: Arm 1: Big White Wall
Participants allocated to receive six months free access to the Big White Wall website [8] were invited to create a user profile using a pseudonym which was linked to the trial identification to which they had been assigned within the study website. They had to do this within 14 days of being randomised. Participants were able to access any part of the BWW site (apart from the option of personalised therapy or counselling sessions which have to be prescribed by a clinician i.e. not offered direct to the general public), and interact with other users within the boundaries of the site's House Rules. Anonymised records of logins, time on site, interactions and page categories were recorded by BWW on behalf of the study team.
Randomisation: Arm 2: Participants allocated to Moodzone
Participants were directed to the Moodzone area of the NHS Choices website [9]. Participants were able to access all the available material on mental health including depression and anxiety. We did not have records of time on site or use of the site. NHS MZ access was used as the control digital resource so all participants were offered some help for their problems with depression or anxiety but this control group did not have access to moderated, anonymised peer social support."
There were no co-interventions.

6a) Completely defined pre-specified primary and secondary outcome measures, including how and when they were assessed

Does your paper address CONSORT subitem 6a? *

Copy and paste relevant sections from the manuscript (include quotes in quotation marks "like this" to indicate direct quotes from your manuscript), or elaborate on this item by providing additional information not in the ms, or briefly explain why the item is not applicable/relevant for your study

"Primary Outcome Measure:
1. Change in self-rated well-being from baseline and 6 weeks after baseline using the 14-item Warwick-Edinburgh Mental Well-Being Scale (WEMWBS) [28].
Secondary Outcomes Measures:
1. Well-being at 3, 12 and 26 weeks using the WEMWBS.
2. 7-Item Generalised Anxiety Disorder Scale (GAD-7) [25] completed as part of eligibility at baseline and then at 3, 6, 12 and 26 weeks, as a measure of anxiety severity .
3. 9-Item Patient Health Questionnaire (PHQ-9) [26] completed as part of eligibility at baseline and then at 3, 6, 12 and 26 weeks, as a measure of depression severity.
4. Social function on the 8-item Work and Social Adjustment Scale (WSAS) [29], a measure of function, completed at baseline,3, 6, 12 and 26 weeks."

6a-i) Online questionnaires: describe if they were validated for online use and apply CHERRIES items to describe how the questionnaires were designed/deployed

If outcomes were obtained through online questionnaires, describe if they were validated for online use and apply CHERRIES items to describe how the questionnaires were designed/deployed [9].

	1	2	3	4	5	
subitem not at all important	○	○	○	○	◉	essential

Clear selection

Does your paper address subitem 6a-i?

Copy and paste relevant sections from manuscript text

These are all well-known outcome measures that have evidence of validity, reliability and sensitivity to change. None of them were developed for on-line use but all of them have been frequently used in other on-line trials and on line clinical practice

6a-ii) Describe whether and how “use” (including intensity of use/dosage) was defined/measured/monitored

Describe whether and how “use” (including intensity of use/dosage) was defined/measured/monitored (logins, logfile analysis, etc.). Use/adoption metrics are important process outcomes that should be reported in any ehealth trial.

	1	2	3	4	5	
subitem not at all important	○	○	○	○	◉	essential

Clear selection

Does your paper address subitem 6a-ii?

Copy and paste relevant sections from manuscript text

"Primary Outcome Measure:
1. Change in self-rated well-being from baseline and 6 weeks after baseline using the 14-item Warwick-Edinburgh Mental Well-Being Scale (WEMWBS) [28].
Secondary Outcomes Measures:
1. Well-being at 3, 12 and 26 weeks using the WEMWBS.
2. 7-Item Generalised Anxiety Disorder Scale (GAD-7) [25] completed as part of eligibility at baseline and then at 3, 6, 12 and 26 weeks, as a measure of anxiety severity .
3. 9-Item Patient Health Questionnaire (PHQ-9) [26] completed as part of eligibility at baseline and then at 3, 6, 12 and 26 weeks, as a measure of depression severity.
4. Social function on the 8-item Work and Social Adjustment Scale (WSAS) [29], a measure of function, completed at baseline,3, 6, 12 and 26 weeks."

6a-iii) Describe whether, how, and when qualitative feedback from participants was obtained

Describe whether, how, and when qualitative feedback from participants was obtained (e.g., through emails, feedback forms, interviews, focus groups).

	1	2	3	4	5	
subitem not at all important	○	○	○	○	●	essential

Clear selection

Does your paper address subitem 6a-iii?

Copy and paste relevant sections from manuscript text

"We asked for survey feedback at baseline and every follow up and also received email and telephone feedback when participants wished to contact the research team".
We have also performed qualitative interviews and analysed this data that we will separately report. It confirms the findings reported on barriers to recruitment and retention to the study and intervention but not reported her because of lack of space.

6b) Any changes to trial outcomes after the trial commenced, with reasons

Does your paper address CONSORT subitem 6b? *

Copy and paste relevant sections from the manuscript (include quotes in quotation marks "like this" to indicate direct quotes from your manuscript), or elaborate on this item by providing additional information not in the ms, or briefly explain why the item is not applicable/relevant for your study

"Feedback left by the first 50 participants suggested they disliked the intrusiveness and length of some of the measures and the assessments at baseline. One participant withdrew from the study for this reason. Therefore, compared to our protocol [24], we omitted the 12-item medical outcomes study short form health survey version 2.0 (SF-12v2) [34] at all time points and only carried out the economic resource proforma [31] at baseline. At baseline the number of questions asked fell from 92 to 80, and at each follow up time point from 50 to 38."

7a) How sample size was determined

NPT: When applicable, details of whether and how the clustering by care provides or centers was addressed

7a-i) Describe whether and how expected attrition was taken into account when calculating the sample size

Describe whether and how expected attrition was taken into account when calculating the sample size.

	1	2	3	4	5	
subitem not at all important	○	○	○	○	●	essential

Clear selection

Does your paper address subitem 7a-i?

Copy and paste relevant sections from manuscript title (include quotes in quotation marks "like this" to indicate direct quotes from your manuscript), or elaborate on this item by providing additional information not in the ms, or briefly explain why the item is not applicable/relevant for your study

"The sample size calculation and justification was outlined in detail in our protocol paper [24]. To detect a 3 point (SD=12) minimal clinically important difference for adults on the 14-item WEMWBS [35] at a 0.05 significance level with 90% power, 676 patients were needed. After adjusting for a 50% attrition rate at 6 weeks [36], a total of 1352 participants was required for our RCT."

7b) When applicable, explanation of any interim analyses and stopping guidelines

Does your paper address CONSORT subitem 7b? *

Copy and paste relevant sections from the manuscript (include quotes in quotation marks "like this" to indicate direct quotes from your manuscript), or elaborate on this item by providing additional information not in the ms, or briefly explain why the item is not applicable/relevant for your study

No interim analysis reported and no stopping guidelines developed.

8a) Method used to generate the random allocation sequence

NPT: When applicable, how care providers were allocated to each trial group

Does your paper address CONSORT subitem 8a? *

Copy and paste relevant sections from the manuscript (include quotes in quotation marks "like this" to indicate direct quotes from your manuscript), or elaborate on this item by providing additional information not in the ms, or briefly explain why the item is not applicable/relevant for your study

"The treatment to which a participant was assigned was determined by a computer generated pseudo-random code using random permuted blocks of varying size by a randomisation system embedded within the website. No stratification or minimisation was used."

8b) Type of randomisation; details of any restriction (such as blocking and block size)

Does your paper address CONSORT subitem 8b? *

Copy and paste relevant sections from the manuscript (include quotes in quotation marks "like this" to indicate direct quotes from your manuscript), or elaborate on this item by providing additional information not in the ms, or briefly explain why the item is not applicable/relevant for your study

"The treatment to which a participant was assigned was determined by a computer generated pseudo-random code using random permuted blocks of varying size by a randomisation system embedded within the website. No stratification or minimisation was used."

9) Mechanism used to implement the random allocation sequence (such as sequentially numbered containers), describing any steps taken to conceal the sequence until interventions were assigned

Does your paper address CONSORT subitem 9? *

Copy and paste relevant sections from the manuscript (include quotes in quotation marks "like this" to indicate direct quotes from your manuscript), or elaborate on this item by providing additional information not in the ms, or briefly explain why the item is not applicable/relevant for your study

"The treatment to which a participant was assigned was determined by a computer generated pseudo-random code using random permuted blocks of varying size by a randomisation system embedded within the website. No stratification or minimisation was used."
"The second stage of the study was a single-blind, randomised controlled feasibility trial (RCT) using a fully automated bespoke study website. Full details are available in the published protocol [24]. Eligible participants self-referred and were recruited through the study website following the public health campaign. Consenting participants were allocated at random to receive either six months free access to BWW or signposted to the NHS Moodzone (MZ) website."
"

10) Who generated the random allocation sequence, who enrolled participants, and who assigned participants to interventions

Does your paper address CONSORT subitem 10? *

Copy and paste relevant sections from the manuscript (include quotes in quotation marks "like this" to indicate direct quotes from your manuscript), or elaborate on this item by providing additional information not in the ms, or briefly explain why the item is not applicable/relevant for your study

"Potential participants from the county of Nottinghamshire self-referred to the study and their eligibility was assessed by an automated digital programme on the study website."
"The treatment to which a participant was assigned was determined by a computer generated pseudo-random code using random permuted blocks of varying size by a randomisation system embedded within the website. No stratification or minimisation was used."
"The second stage of the study was a single-blind, randomised controlled feasibility trial (RCT) using a fully automated bespoke study website. Full details are available in the published protocol [24]. Eligible participants self-referred and were recruited through the study website following the public health campaign. Consenting participants were allocated at random to receive either six months free access to BWW or signposted to the NHS Moodzone (MZ) website."

11a) If done, who was blinded after assignment to interventions (for example, participants, care providers, those assessing outcomes) and how

NPT: Whether or not administering co-interventions were blinded to group assignment

11a-i) Specify who was blinded, and who wasn’t

Specify who was blinded, and who wasn’t. Usually, in web-based trials it is not possible to blind the participants [1, 3] (this should be clearly acknowledged), but it may be possible to blind outcome assessors, those doing data analysis or those administering co-interventions (if any).

	1	2	3	4	5	
subitem not at all important	○	○	○	○	◉	essential

Clear selection

Does your paper address subitem 11a-i? *

Copy and paste relevant sections from the manuscript (include quotes in quotation marks "like this" to indicate direct quotes from your manuscript), or elaborate on this item by providing additional information not in the ms, or briefly explain why the item is not applicable/relevant for your study

Fully automated trial of 2 fully automated trial interventions. Data checked and downloaded by trial manager. Analysis of outcomes conducted by a statistician fully blinded to all trial processes and meetings until data was locked. All of this has ben repeatedly described above.

11a-ii) Discuss e.g., whether participants knew which intervention was the "intervention of interest" and which one was the "comparator"

Informed consent procedures (4a-ii) can create biases and certain expectations - discuss e.g., whether participants knew which intervention was the "intervention of interest" and which one was the "comparator".

	1	2	3	4	5	
subitem not at all important	○	○	○	○	◉	essential

Clear selection

Does your paper address subitem 11a-ii?

Copy and paste relevant sections from the manuscript (include quotes in quotation marks "like this" to indicate direct quotes from your manuscript), or elaborate on this item by providing additional information not in the ms, or briefly explain why the item is not applicable/relevant for your study

Description of interventions already entered several times shows that participants were not blinded to the intervention they received (which would be impossible in this instance).

11b) If relevant, description of the similarity of interventions

(this item is usually not relevant for ehealth trials as it refers to similarity of a placebo or sham intervention to a active medication/intervention)

Does your paper address CONSORT subitem 11b? *

Copy and paste relevant sections from the manuscript (include quotes in quotation marks "like this" to indicate direct quotes from your manuscript), or elaborate on this item by providing additional information not in the ms, or briefly explain why the item is not applicable/relevant for your study

"Randomisation: Arm 1: Big White Wall
Participants allocated to receive six months free access to the Big White Wall website [8] were invited to create a user profile using a pseudonym which was linked to the trial identification to which they had been assigned within the study website. They had to do this within 14 days of being randomised. Participants were able to access any part of the BWW site (apart from the option of personalised therapy or counselling sessions which have to be prescribed by a clinician i.e. not offered direct to the general public), and interact with other users within the boundaries of the site’s House Rules. Anonymised records of logins, time on site, interactions and page categories were recorded by BWW on behalf of the study team.
Randomisation: Arm 2: Participants allocated to Moodzone
Participants were directed to the Moodzone area of the NHS Choices website [9]. Participants were able to access all the available material on mental health including depression and anxiety. We did not have records of time on site or use of the site. NHS MZ access was used as the control digital resource so all participants were offered some help for their problems with depression or anxiety but this control group did not have access to moderated, anonymised peer social support."

12a) Statistical methods used to compare groups for primary and secondary outcomes

NPT: When applicable, details of whether and how the clustering by care providers or centers was addressed

Does your paper address CONSORT subitem 12a? *

Copy and paste relevant sections from the manuscript (include quotes in quotation marks "like this" to indicate direct quotes from your manuscript), or elaborate on this item by providing additional information not in the ms, or briefly explain why the item is not applicable/relevant for your study

"Since all outcome scores were repeatedly measured at baseline, 3, 6, 12 and 26 weeks, multilevel modelling was performed to quantify the treatment effect with participant as a level 2 unit and baseline, treatment arm, follow up time and interaction of arm×time as a covariate. Missing outcome values were investigated and imputed for all outcomes under the missing at random assumption with 100 datasets imputed for data analysis. REALCOME and STATA 16 were used to impute missingness. Similar models were conducted on observed values to check the robustness of treatment effect estimates sensitive to missingness influence."

12a-i) Imputation techniques to deal with attrition / missing values

Imputation techniques to deal with attrition / missing values: Not all participants will use the intervention/comparator as intended and attrition is typically high in ehealth trials. Specify how participants who did not use the application or dropped out from the trial were treated in the statistical analysis (a complete case analysis is strongly discouraged, and simple imputation techniques such as LOCF may also be problematic [4]).

	1	2	3	4	5	
subitem not at all important	○	○	○	○	◉	essential

Clear selection

Does your paper address subitem 12a-i? *

Copy and paste relevant sections from the manuscript (include quotes in quotation marks "like this" to indicate direct quotes from your manuscript), or elaborate on this item by providing additional information not in the ms, or briefly explain why the item is not applicable/relevant for your study

"Since all outcome scores were repeatedly measured at baseline, 3, 6, 12 and 26 weeks, multilevel modelling was performed to quantify the treatment effect with participant as a level 2 unit and baseline, treatment arm, follow up time and interaction of arm×time as a covariate. Missing outcome values were investigated and imputed for all outcomes under the missing at random assumption with 100 datasets imputed for data analysis. REALCOME and STATA 16 were used to impute missingness. Similar models were conducted on observed values to check the robustness of treatment effect estimates sensitive to missingness influence."

12b) Methods for additional analyses, such as subgroup analyses and adjusted analyses

Does your paper address CONSORT subitem 12b? *

Copy and paste relevant sections from the manuscript (include quotes in quotation marks "like this" to indicate direct quotes from your manuscript), or elaborate on this item by providing additional information not in the ms, or briefly explain why the item is not applicable/relevant for your study

"Since all outcome scores were repeatedly measured at baseline, 3, 6, 12 and 26 weeks, multilevel modelling was performed to quantify the treatment effect with participant as a level 2 unit and baseline, treatment arm, follow up time and interaction of arm×time as a covariate. Missing outcome values were investigated and imputed for all outcomes under the missing at random assumption with 100 datasets imputed for data analysis. REALCOME and STATA 16 were used to impute missingness. Similar models were conducted on observed values to check the robustness of treatment effect estimates sensitive to missingness influence."
No subgroup analysis.

X26) REB/IRB Approval and Ethical Considerations [recommended as subheading under "Methods"] (not a CONSORT item)

X26-i) Comment on ethics committee approval

	1	2	3	4	5	
subitem not at all important	○	○	○	○	◉	essential

Clear selection

Does your paper address subitem X26-i?

Copy and paste relevant sections from the manuscript (include quotes in quotation marks "like this" to indicate direct quotes from your manuscript), or elaborate on this item by providing additional information not in the ms, or briefly explain why the item is not applicable/relevant for your study

Ethical approval was granted by the Local Research Ethics Committee (REC 16/EM/0204) and final approval received from the UK Health Research Authority.

x26-ii) Outline informed consent procedures

Outline informed consent procedures e.g., if consent was obtained offline or online (how? Checkbox, etc.?), and what information was provided (see 4a-ii). See [6] for some items to be included in informed consent documents.

	1	2	3	4	5	
subitem not at all important	○	○	○	○	◉	essential

Clear selection

Does your paper address subitem X26-ii?

Copy and paste relevant sections from the manuscript (include quotes in quotation marks "like this" to indicate direct quotes from your manuscript), or elaborate on this item by providing additional information not in the ms, or briefly explain why the item is not applicable/relevant for your study

"Information for participants and the associated consent forms were provided electronically within the study website. Participants who wished to discuss the study could email and telephone the study team if they had any further questions before consenting to the study. An email confirming consent was sent to each participant once they had fully enrolled."

X26-iii) Safety and security procedures

Safety and security procedures, incl. privacy considerations, and any steps taken to reduce the likelihood or detection of harm (e.g., education and training, availability of a hotline)

	1	2	3	4	5	
subitem not at all important	○	○	○	○	◉	essential

Clear selection

Does your paper address subitem X26-iii?

Copy and paste relevant sections from the manuscript (include quotes in quotation marks "like this" to indicate direct quotes from your manuscript), or elaborate on this item by providing additional information not in the ms, or briefly explain why the item is not applicable/relevant for your study

"Participants who were ineligible for the trial because they scored in the severe range on the PHQ-9, or scored 2 or 3 on the suicide item of the PHQ-9, were provided with an opportunity to request that the study team inform their GP, mental health care team or carer of their current mood state. This was in line with other digital studies [27] and was required by our national ethics committee. If the request was not completed, the study team followed up via email asking if they would like the team to inform their GP or care team. We followed up participants on one occasion."

RESULTS

13a) For each group, the numbers of participants who were randomly assigned, received intended treatment, and were analysed for the primary outcome

NPT: The number of care providers or centers performing the intervention in each group and the number of patients treated by each care provider in each center

Does your paper address CONSORT subitem 13a? *

Copy and paste relevant sections from the manuscript (include quotes in quotation marks "like this" to indicate direct quotes from your manuscript), or elaborate on this item by providing additional information not in the ms, or briefly explain why the item is not applicable/relevant for your study

See Figure 1.

13b) For each group, losses and exclusions after randomisation, together with reasons

Does your paper address CONSORT subitem 13b? (NOTE: Preferably, this is shown in a CONSORT flow diagram) *

Copy and paste relevant sections from the manuscript (include quotes in quotation marks "like this" to indicate direct quotes from your manuscript), or elaborate on this item by providing additional information not in the ms, or briefly explain why the item is not applicable/relevant for your study

See Figure 1

13b-i) Attrition diagram

Strongly recommended: An attrition diagram (e.g., proportion of participants still logging in or using the intervention/comparator in each group plotted over time, similar to a survival curve) or other figures or tables demonstrating usage/dose/engagement.

	1	2	3	4	5	
subitem not at all important	○	○	●	○	○	essential

Clear selection

Does your paper address subitem 13b-i?

Copy and paste relevant sections from the manuscript or cite the figure number if applicable (include quotes in quotation marks "like this" to indicate direct quotes from your manuscript), or elaborate on this item by providing additional information not in the ms, or briefly explain why the item is not applicable/relevant for your study

"Of the 393 participants randomised to BWW, 225 (57.3%) registered to access BWW and 165 (42.5) accessed it on more than one occasion. No participation data are available for the MZ website." This is an as needed intervention so an attrition diagram is not required.

14a) Dates defining the periods of recruitment and follow-up

Does your paper address CONSORT subitem 14a? *

Copy and paste relevant sections from the manuscript (include quotes in quotation marks "like this" to indicate direct quotes from your manuscript), or elaborate on this item by providing additional information not in the ms, or briefly explain why the item is not applicable/relevant for your study

Figure 1 shows that there were 6483 visitors to the study website (14 per day) over 18 months of recruitment from September 16th 2016 to May 30th 2018. Follow up was for a maximum of 26 weeks for all participants (automated)

14a-i) Indicate if critical "secular events" fell into the study period

Indicate if critical "secular events" fell into the study period, e.g., significant changes in Internet resources available or "changes in computer hardware or Internet delivery resources"

	1	2	3	4	5	
subitem not at all important	○	○	●	○	○	essential

Clear selection

Does your paper address subitem 14a-i?

Copy and paste relevant sections from the manuscript (include quotes in quotation marks "like this" to indicate direct quotes from your manuscript), or elaborate on this item by providing additional information not in the ms, or briefly explain why the item is not applicable/relevant for your study

None occurred.

14b) Why the trial ended or was stopped (early)

Does your paper address CONSORT subitem 14b? *

Copy and paste relevant sections from the manuscript (include quotes in quotation marks "like this" to indicate direct quotes from your manuscript), or elaborate on this item by providing additional information not in the ms, or briefly explain why the item is not applicable/relevant for your study

It was not stopped early.

15) A table showing baseline demographic and clinical characteristics for each group

NPT: When applicable, a description of care providers (case volume, qualification, expertise, etc.) and centers (volume) in each group

Does your paper address CONSORT subitem 15? *

Copy and paste relevant sections from the manuscript (include quotes in quotation marks "like this" to indicate direct quotes from your manuscript), or elaborate on this item by providing additional information not in the ms, or briefly explain why the item is not applicable/relevant for your study

See Table 2.

15-i) Report demographics associated with digital divide issues

In ehealth trials it is particularly important to report demographics associated with digital divide issues, such as age, education, gender, social-economic status, computer/Internet/ehealth literacy of the participants, if known.

	1	2	3	4	5	
subitem not at all important	○	○	○	○	◉	essential

Clear selection

Does your paper address subitem 15-i? *

Copy and paste relevant sections from the manuscript (include quotes in quotation marks "like this" to indicate direct quotes from your manuscript), or elaborate on this item by providing additional information not in the ms, or briefly explain why the item is not applicable/relevant for your study

Table 2. No measure of computer literacy recorded.

16) For each group, number of participants (denominator) included in each analysis and whether the analysis was by original assigned groups

16-i) Report multiple “denominators” and provide definitions

Report multiple “denominators” and provide definitions: Report N’s (and effect sizes) “across a range of study participation [and use] thresholds” [1], e.g., N exposed, N consented, N used more than x times, N used more than y weeks, N participants “used” the intervention/comparator at specific pre-defined time points of interest (in absolute and relative numbers per group). Always clearly define “use” of the intervention.

	1	2	3	4	5	
subitem not at all important	○	○	○	○	◉	essential

Clear selection

Does your paper address subitem 16-i? *

Copy and paste relevant sections from the manuscript (include quotes in quotation marks "like this" to indicate direct quotes from your manuscript), or elaborate on this item by providing additional information not in the ms, or briefly explain why the item is not applicable/relevant for your study

Tables 2 to 5.

16-ii) Primary analysis should be intent-to-treat

Primary analysis should be intent-to-treat, secondary analyses could include comparing only “users”, with the appropriate caveats that this is no longer a randomized sample (see 18-i).

	1	2	3	4	5	
subitem not at all important	○	○	○	○	◉	essential

Clear selection

Does your paper address subitem 16-ii?

Copy and paste relevant sections from the manuscript (include quotes in quotation marks "like this" to indicate direct quotes from your manuscript), or elaborate on this item by providing additional information not in the ms, or briefly explain why the item is not applicable/relevant for your study

Table 5 and statistical method (quote already provided).

17a) For each primary and secondary outcome, results for each group, and the estimated effect size and its precision (such as 95% confidence interval)

Does your paper address CONSORT subitem 17a? *

Copy and paste relevant sections from the manuscript (include quotes in quotation marks "like this" to indicate direct quotes from your manuscript), or elaborate on this item by providing additional information not in the ms, or briefly explain why the item is not applicable/relevant for your study

Table 5

17a-i) Presentation of process outcomes such as metrics of use and intensity of use

In addition to primary/secondary (clinical) outcomes, the presentation of process outcomes such as metrics of use and intensity of use (dose, exposure) and their operational definitions is critical. This does not only refer to metrics of attrition (13-b) (often a binary variable), but also to more continuous exposure metrics such as "average session length". These must be accompanied by a technical description how a metric like a "session" is defined (e.g., timeout after idle time) [1] (report under item 6a).

	1	2	3	4	5	
subitem not at all important	○	○	○	○	●	essential

Clear selection

Does your paper address subitem 17a-i?

Copy and paste relevant sections from the manuscript (include quotes in quotation marks "like this" to indicate direct quotes from your manuscript), or elaborate on this item by providing additional information not in the ms, or briefly explain why the item is not applicable/relevant for your study

Figure 1.
"Of the 393 participants randomised to BWW, 225 (57.3%) registered to access BWW and 165 (42.5) accessed it on more than one occasion. No participation data are available for the MZ website."
More complete data on interactive conversations on Big White Wall will be reported separately.

17b) For binary outcomes, presentation of both absolute and relative effect sizes is recommended

Does your paper address CONSORT subitem 17b? *

Copy and paste relevant sections from the manuscript (include quotes in quotation marks "like this" to indicate direct quotes from your manuscript), or elaborate on this item by providing additional information not in the ms, or briefly explain why the item is not applicable/relevant for your study

No binary outcomes reported.

18) Results of any other analyses performed, including subgroup analyses and adjusted analyses, distinguishing pre-specified from exploratory

Does your paper address CONSORT subitem 18? *

Copy and paste relevant sections from the manuscript (include quotes in quotation marks "like this" to indicate direct quotes from your manuscript), or elaborate on this item by providing additional information not in the ms, or briefly explain why the item is not applicable/relevant for your study

No subgroup analysis.

18-i) Subgroup analysis of comparing only users

A subgroup analysis of comparing only users is not uncommon in ehealth trials, but if done, it must be stressed that this is a self-selected sample and no longer an unbiased sample from a randomized trial (see 16-iii).

	1	2	3	4	5	
subitem not at all important	○	○	●	○	○	essential

Clear selection

Does your paper address subitem 18-i?

Copy and paste relevant sections from the manuscript (include quotes in quotation marks "like this" to indicate direct quotes from your manuscript), or elaborate on this item by providing additional information not in the ms, or briefly explain why the item is not applicable/relevant for your study

No subgroup analysis necessary since retention rates in the study are so low that the results would be misleading.

19) All important harms or unintended effects in each group

(for specific guidance see CONSORT for harms)

Does your paper address CONSORT subitem 19? *

Copy and paste relevant sections from the manuscript (include quotes in quotation marks "like this" to indicate direct quotes from your manuscript), or elaborate on this item by providing additional information not in the ms, or briefly explain why the item is not applicable/relevant for your study

No harms or unintended consequences from trial reported. Harms reported of not being permitted to participate in the trial.
"Potential participants with more severe depression who were trying to take part but were turned away by the automated eligibility criteria on the REBOOT website, expressed disappointment, frustration and a sense of exclusion made apparent through a number of complaints to the study email account."
"

19-i) Include privacy breaches, technical problems

Include privacy breaches, technical problems. This does not only include physical "harm" to participants, but also incidents such as perceived or real privacy breaches [1], technical problems, and other unexpected/unintended incidents. "Unintended effects" also includes unintended positive effects [2].

	1	2	3	4	5	
subitem not at all important	○	○	○	○	○	essential

Does your paper address subitem 19-i?

Copy and paste relevant sections from the manuscript (include quotes in quotation marks "like this" to indicate direct quotes from your manuscript), or elaborate on this item by providing additional information not in the ms, or briefly explain why the item is not applicable/relevant for your study

No privacy breaches.
"Although people contacted the research team over technical problems, contact with the research team usually led to a greater engagement by those participants in follow up. The study experienced technical issues, such as website downtime, problems with progression through the site and errors within the measures, which may have deterred completion of some measures and retention in the study."

19-ii) Include qualitative feedback from participants or observations from staff/researchers

Include qualitative feedback from participants or observations from staff/researchers, if available, on strengths and shortcomings of the application, especially if they point to unintended/unexpected effects or uses. This includes (if available) reasons for why people did or did not use the application as intended by the developers.

	1	2	3	4	5	
subitem not at all important	○	○	○	○	○	essential

Does your paper address subitem 19-ii?

Copy and paste relevant sections from the manuscript (include quotes in quotation marks "like this" to indicate direct quotes from your manuscript), or elaborate on this item by providing additional information not in the ms, or briefly explain why the item is not applicable/relevant for your study

"We collected the following feedback on barriers to participation and retention to the study from randomised participants, those who had considered participating, and those who refused to participate but left comments for the research team by email, text or survey :

1. Lack of personal interaction from the research team was disliked. The study was set up to be automated so that a member of the public could participate in the trial as they might do with other online applications, only seeking technical support if and when they need it. However, this meant a lack of a personal connection and engagement with the research team and participants described this as contributing to a lack of obligation to complete the study measures and participate in follow up time points.
2. Potential participants with more severe depression who were trying to take part but were turned away by the automated eligibility criteria on the REBOOT website, expressed disappointment, frustration and a sense of exclusion made apparent through a number of complaints to the study email account.
3. Lack of technical support. Although people contacted the research team over technical problems, contact with the research team usually led to a greater engagement by those participants in follow up. The study experienced technical issues, such as website downtime, problems with progression through the site and errors within the measures, which may have deterred completion of some measures and retention in the study."

DISCUSSION

22) Interpretation consistent with results, balancing benefits and harms, and considering other relevant evidence

NPT: In addition, take into account the choice of the comparator, lack of or partial blinding, and unequal expertise of care providers or centers in each group

22-i) Restate study questions and summarize the answers suggested by the data, starting with primary outcomes and process outcomes (use)

Restate study questions and summarize the answers suggested by the data, starting with primary outcomes and process outcomes (use).

	1	2	3	4	5	
subitem not at all important	○	○	○	○	◉	essential

Clear selection

Does your paper address subitem 22-i? *

Copy and paste relevant sections from the manuscript (include quotes in quotation marks "like this" to indicate direct quotes from your manuscript), or elaborate on this item by providing additional information not in the ms, or briefly explain why the item is not applicable/relevant for your study

"Overall, we found that running a fully automated trial of an online intervention was very challenging. Exclusion criteria, exclusively online enrolment and measurement, restricted full enrolment and participation for complete follow-up. We recruited and randomised only 790 (58.4%) of those who expressed interest and were eligible, despite a considerable amount of effort by the research team using traditional advertising, internet and social media, health service, other public service and third sector contacts."
"Only 16.6% of the enrolled sample provided primary outcome data at 6 weeks. As a result the study did not have the statistical power to determine whether or not BWW was more clinically effective than MZ."

22-ii) Highlight unanswered new questions, suggest future research

Highlight unanswered new questions, suggest future research.

	1	2	3	4	5	
subitem not at all important	○	○	○	○	◉	essential

Clear selection

Does your paper address subitem 22-ii?

Copy and paste relevant sections from the manuscript (include quotes in quotation marks "like this" to indicate direct quotes from your manuscript), or elaborate on this item by providing additional information not in the ms, or briefly explain why the item is not applicable/relevant for your study

"We failed to recruit enough males, older participants, people from BAME backgrounds, people without any educational qualifications and people in more rural areas, each of whom may require a combination of different strategies for enrolment.",
"Our data suggests that a process of interaction with a human or possibly a virtual human is required to ensure fully informed consent and commitment to the study."

20) Trial limitations, addressing sources of potential bias, imprecision, and, if relevant, multiplicity of analyses

20-i) Typical limitations in ehealth trials

Typical limitations in ehealth trials: Participants in ehealth trials are rarely blinded. Ehealth trials often look at a multiplicity of outcomes, increasing risk for a Type I error. Discuss biases due to non-use of the intervention/usability issues, biases through informed consent procedures, unexpected events.

	1	2	3	4	5	
subitem not at all important	○	○	○	○	◉	essential

Clear selection

Does your paper address subitem 20-i? *

Copy and paste relevant sections from the manuscript (include quotes in quotation marks "like this" to indicate direct quotes from your manuscript), or elaborate on this item by providing additional information not in the ms, or briefly explain why the item is not applicable/relevant for your study

The key issues for this automated RCT are around poor retention and recruitment which are extensively discussed.

21) Generalisability (external validity, applicability) of the trial findings

NPT: External validity of the trial findings according to the intervention, comparators, patients, and care providers or centers involved in the trial

21-i) Generalizability to other populations

Generalizability to other populations: In particular, discuss generalizability to a general Internet population, outside of a RCT setting, and general patient population, including applicability of the study results for other organizations

	1	2	3	4	5	
subitem not at all important	○	○	○	○	●	essential

Clear selection

Does your paper address subitem 21-i?

Copy and paste relevant sections from the manuscript (include quotes in quotation marks "like this" to indicate direct quotes from your manuscript), or elaborate on this item by providing additional information not in the ms, or briefly explain why the item is not applicable/relevant for your study

"We recruited an overwhelmingly female, white ethnic, and educated sample who were mostly currently in work or education. Around half of those enrolled were not in contact with any health service in the preceding three months. A core aim of providing digital mental health approaches to reach people who are not in contact with health services was achieved. Younger females are a part of the population with increasing rates of depression and anxiety in the UK [2] and this study suggests that such people might be reached through digital direct to the public services. However, we failed to recruit enough males, older participants, people from BAME backgrounds, people without any educational qualifications and people in more rural areas, each of whom may require a combination of different strategies for enrolment."

21-ii) Discuss if there were elements in the RCT that would be different in a routine application setting

Discuss if there were elements in the RCT that would be different in a routine application setting (e.g., prompts/reminders, more human involvement, training sessions or other co-interventions) and what impact the omission of these elements could have on use, adoption, or outcomes if the intervention is applied outside of a RCT setting.

	1	2	3	4	5	
subitem not at all important	○	○	○	○	◉	essential

Clear selection

Does your paper address subitem 21-ii?

Copy and paste relevant sections from the manuscript (include quotes in quotation marks "like this" to indicate direct quotes from your manuscript), or elaborate on this item by providing additional information not in the ms, or briefly explain why the item is not applicable/relevant for your study

We discuss what would be needed to recruit and retain participants who are not white educated women.
"We recruited an overwhelmingly female, white ethnic, and educated sample who were mostly currently in work or education. Around half of those enrolled were not in contact with any health service in the preceding three months. A core aim of providing digital mental health approaches to reach people who are not in contact with health services was achieved. Younger females are a part of the population with increasing rates of depression and anxiety in the UK [2] and this study suggests that such people might be reached through digital direct to the public services. However, we failed to recruit enough males, older participants, people from BAME backgrounds, people without any educational qualifications and people in more rural areas, each of whom may require a combination of different strategies for enrolment".

OTHER INFORMATION

23) Registration number and name of trial registry

Does your paper address CONSORT subitem 23? *

Copy and paste relevant sections from the manuscript (include quotes in quotation marks "like this" to indicate direct quotes from your manuscript), or elaborate on this item by providing additional information not in the ms, or briefly explain why the item is not applicable/relevant for your study

At the end of the abstract.Trial Registration: International Standard Randomized Controlled Trial Number (ISRCTN) 12673428; http://www.controlled-trials.com/ISRCTN12673428/12673428.

24) Where the full trial protocol can be accessed, if available

Does your paper address CONSORT subitem 24? *

Cite a Multimedia Appendix, other reference, or copy and paste relevant sections from the manuscript (include quotes in quotation marks "like this" to indicate direct quotes from your manuscript), or elaborate on this item by providing additional information not in the ms, or briefly explain why the item is not applicable/relevant for your study

Reference 24. 24. Kaylor-Hughes CJ, Rawsthorne M, Coulson NS, Simpson S, Simons L, Guo B, James M, Moran P, Simpson J, Hollis C, Avery AJ, Tata LJ, Williams L; REBOOT Notts Lived Experience Advisory Panel, Morriss RK. Direct to public peer support and e-therapy program versus information to aid self-management of depression and anxiety: Protocol for a randomized controlled trial. JMIR Res Protoc. 2017;6:e231. PMID: 29254909

25) Sources of funding and other support (such as supply of drugs), role of funders

Does your paper address CONSORT subitem 25? *

Copy and paste relevant sections from the manuscript (include quotes in quotation marks "like this" to indicate direct quotes from your manuscript), or elaborate on this item by providing additional information not in the ms, or briefly explain why the item is not applicable/relevant for your study

Reported in acknowledgements.
"Acknowledgements.
This study was funded by National Institute for Health Research (NIHR) Collaborations for Leadership in Applied Health Research and Care East Midlands a and acknowledges the support of the NIHR Clinical Research Network (NIHR CRN) Nottinghamshire Healthcare NHS Foundation Trust Research Delivery Team, and the REBOOT Lived Experience Advisory Panel. RM acknowledges support from the NIHR Applied Research Collaboration East Midlands Nottingham NIHR Biomedical Research Centre and NIHR MindTech MedTech and In-vitro Collaborative. The views expressed in this publication are those of the authors and not necessarily those of the NIHR, the Department of Health and Social Care or Big White Wall."

X27) Conflicts of Interest (not a CONSORT item)

X27-i) State the relation of the study team towards the system being evaluated

In addition to the usual declaration of interests (financial or otherwise), also state the relation of the study team towards the system being evaluated, i.e., state if the authors/evaluators are distinct from or identical with the developers/sponsors of the intervention.

	1	2	3	4	5	
subitem not at all important	○	○	○	○	◉	essential

Clear selection

Does your paper address subitem X27-i?

Copy and paste relevant sections from the manuscript (include quotes in quotation marks "like this" to indicate direct quotes from your manuscript), or elaborate on this item by providing additional information not in the ms, or briefly explain why the item is not applicable/relevant for your study

Statement in manuscript. "The authors report no competing interests."

About the CONSORT EHEALTH checklist

As a result of using this checklist, did you make changes in your manuscript? *

- ○ yes, major changes
- ○ yes, minor changes
- ◉ no

What were the most important changes you made as a result of using this checklist?

None

How much time did you spend on going through the checklist INCLUDING making changes in your manuscript *

2 days.

As a result of using this checklist, do you think your manuscript has improved? *

- ○ yes
- ◉ no
- ○ Other:

Would you like to become involved in the CONSORT EHEALTH group?

This would involve for example becoming involved in participating in a workshop and writing an "Explanation and Elaboration" document

- ○ yes
- ◉ no
- ○ Other:

Clear selection

Any other comments or questions on CONSORT EHEALTH

This was a very repetitive and excessively time-consuming experience which needs to be streamlined and made more efficient. It will deter reporting of trials because of its onerous nature.

STOP - Save this form as PDF before you click submit

To generate a record that you filled in this form, we recommend to generate a PDF of this page (on a Mac, simply select "print" and then select "print as PDF") before you submit it.

When you submit your (revised) paper to JMIR, please upload the PDF as supplementary file.

Don't worry if some text in the textboxes is cut off, as we still have the complete information in our database. Thank you!

Final step: Click submit !

Click submit so we have your answers in our database!

Submit

Never submit passwords through Google Forms.

This content is neither created nor endorsed by Google. Report Abuse - Terms of Service - Privacy Policy

Google Forms
